# Supplementary material for: Atmospheric health burden across the century and the accelerating impact of temperature compared to pollution
Source: Nat Commun. 2024 Oct 30;15:9379. doi: 10.1038/s41467-024-53649-9 (PMC11525551; doi:10.1038/s41467-024-53649-9)
Supplement: Supplementary file 1 — Supplementary Information [file 41467_2024_53649_MOESM1_ESM.pdf]

Supplementary Information :

Atmospheric health burden across the century and the accelerating impact of temperature compared to pollution.

Andrea Pozzer[1,2*], Brendan Steffens[1], Yiannis Proestos[2], Jean Sciare[2], Dimitris Akritidis[1,3], Sourangsu Chowdhury[4], Katrin Burkart[5], Sara Bacer[1]

[1*]Atmospheric Chemistry Department, Max Planck Institute for Chemistry, Hahn-Meitner weg, Mainz, 55128, Germany.
[2]Climate and Atmosphere Research Center, The Cyprus Institute, 20 Konstantinou Kavafi Street, Nicosia, 2121, Cyprus
[3]Department of Meteorology and Climatology, School of Geology, Aristotle University of Thessaloniki, Thessaloniki, 54124, Greece.
[4] CICERO Center for International Climate Research, Oslo, 0349, Norway
[5] Department of Health Metrics Sciences, University of Washington, 15th Ave NE, 3980, Seattle, 98195, Washington, United States.

This PDF file includes:

Supplementary Figures 1 to 9

Supplementary Tables 1 to 4

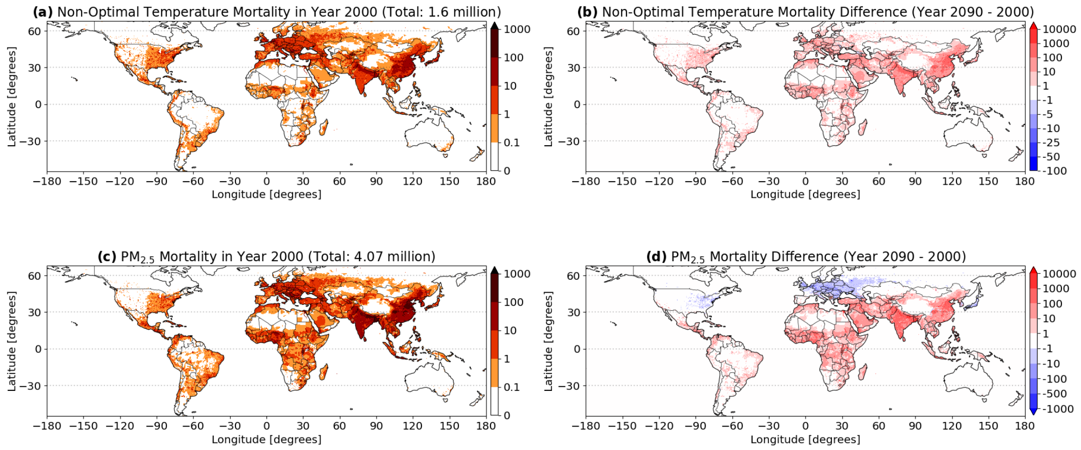

Supplementary Figure 1 Annual mortality for the year 2000 attributable to long-term exposure to non-optimal temperature (a) and to air pollution (c). The differences for the same results by the end of the century in the Shared Socio-economic Pathways SSP1-2.6 scenario are also presented (b) and (d).

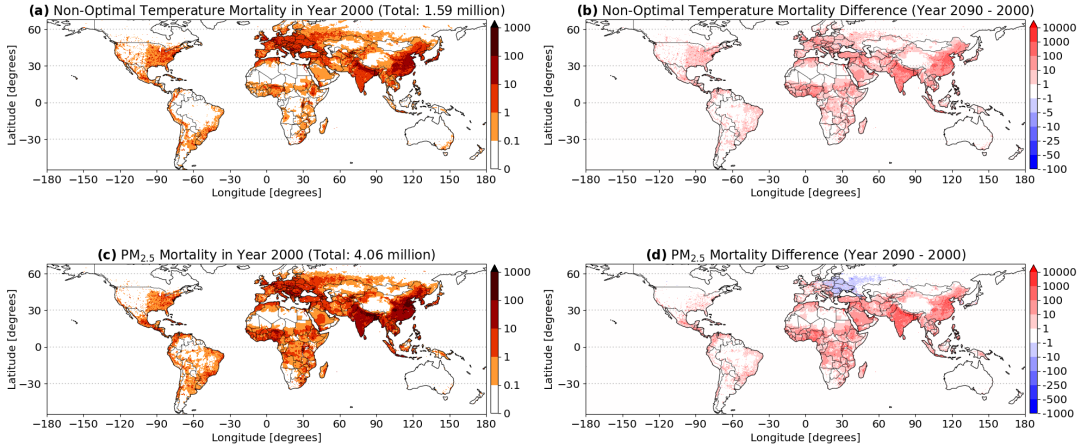

Supplementary Figure 2 Annual mortality for the year 2000 attributable to long-term exposure to non-optimal temperature (a) and to air pollution (c). The differences for the same results by the end of the century in the Shared Socio-economic Pathways SSP5-8.5 scenario are also presented (b) and (d).

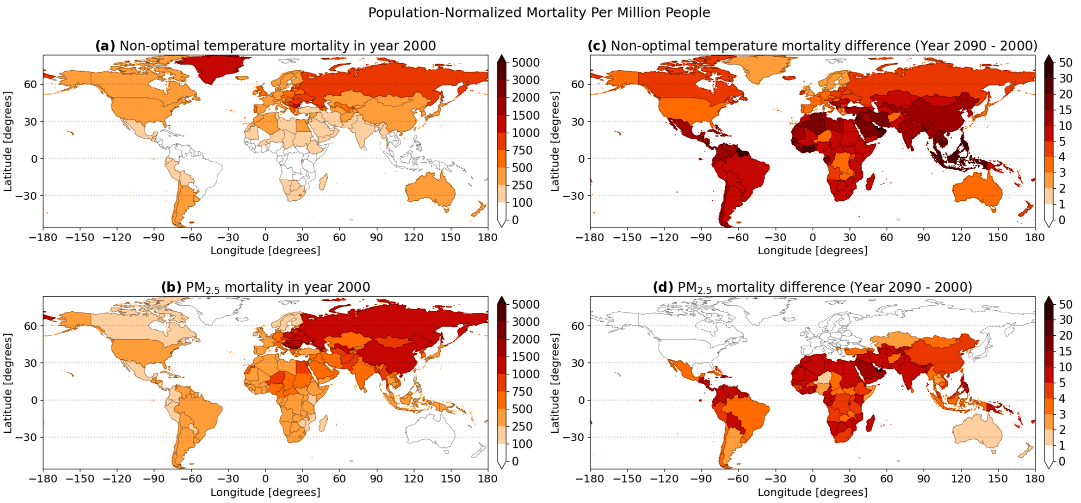

Supplementary Figure 3 Country-level, population-normalized mortality per million people for the year 2000 attributable to long-term exposure to non-optimal temperature (a) and to air pollution (c). The differences for the same results by the end of the century in the Shared Socio-economic Pathways SSP1-2.6 scenario are also presented (b) and (d).

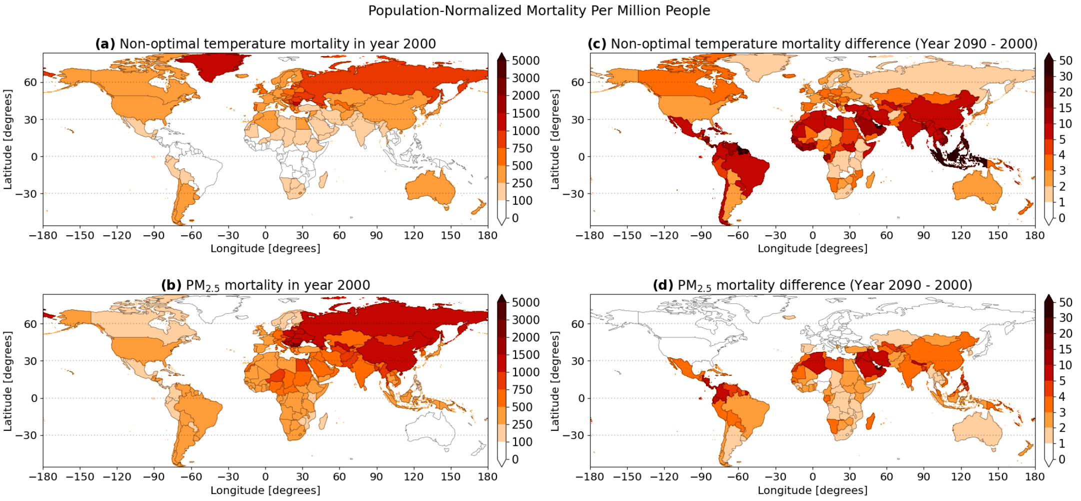

Supplementary Figure 4 Country-level, population-normalized mortality per million people for the year 2000 attributable to long-term exposure to non-optimal temperature (a) and to air pollution (c). The differences for the same results by the end of the century in the Shared Socio-economic Pathways SSP2-4.5 scenario are also presented (b) and (d).

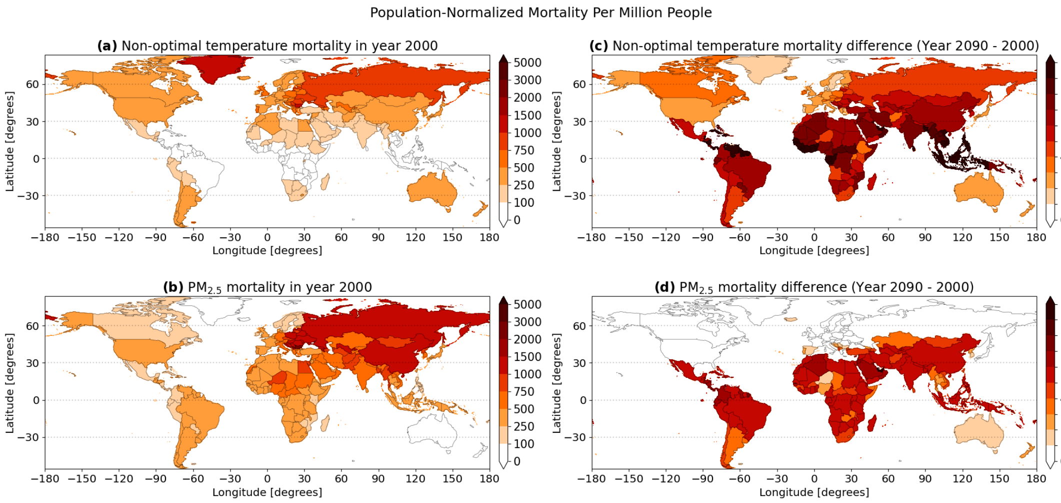

Supplementary Figure 5 Country-level, population-normalized mortality per million people for the year 2000 attributable to long-term exposure to non-optimal temperature (a) and to air pollution (c). The differences for the same results by the end of the century in the Shared Socio-economic Pathways SSP5-8.5 scenario are also presented (b) and (d).

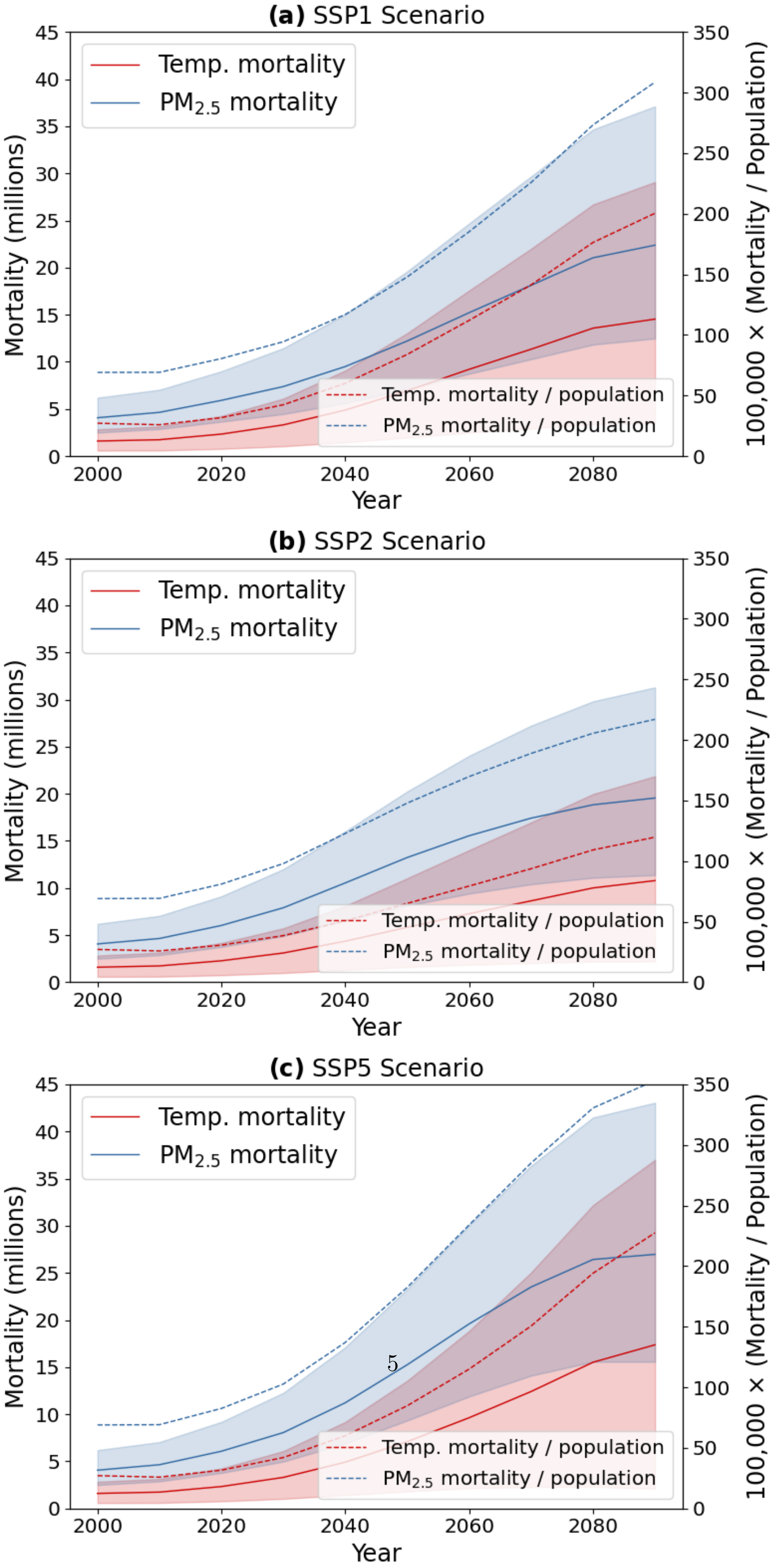

Supplementary Figure 6 Projected mortality as a function of time for the Shared Socio-economic Pathways SSP1-2.6 (a), SSP2-4.5 (b) and the SSP5-8.5 scenario (c). The solid curves, corresponding to the left vertical axes, show the total global mortality (in millions) due to air pollution and non-optimal temperature, with shading indicating the 95% confidence interval. The dashed curves, corresponding to the right axes, show the population-normalized mortality per 100,000 people.

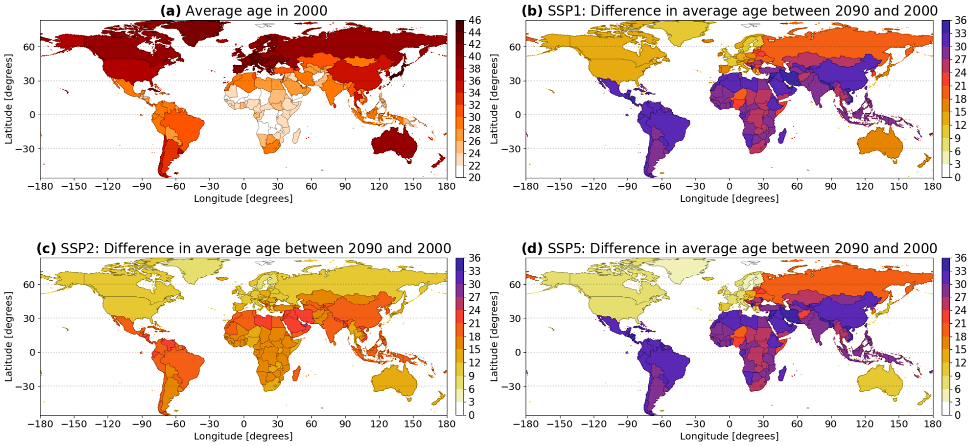

Supplementary Figure 7 Average age by country in 2000 (a), and the difference in average age by 2090 for the Shared Socio-economic Pathways SSP1-2.6 (b), SSP2-4.5 (c) and SSP5-8.5 scenario (d).

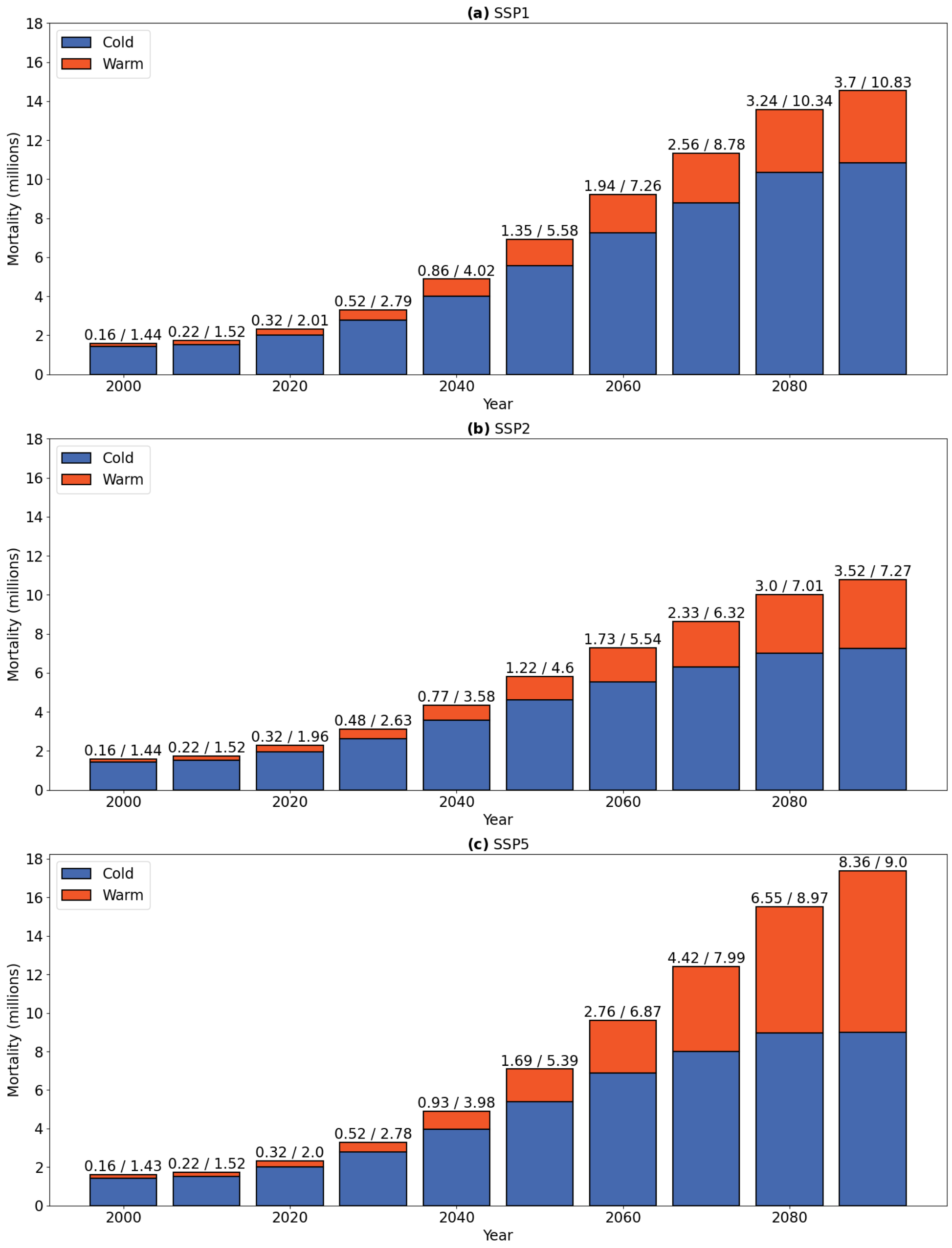

Supplementary Figure 8 Mortality due to exposure to warm and cold temperature for the Shared Socio-economic Pathways SSP1-2.6 (a), SSP2-4.5 (b) and the SSP5-8.5 (c) scenario. For each year, bars are labeled by the total warm / cold mortality in millions.

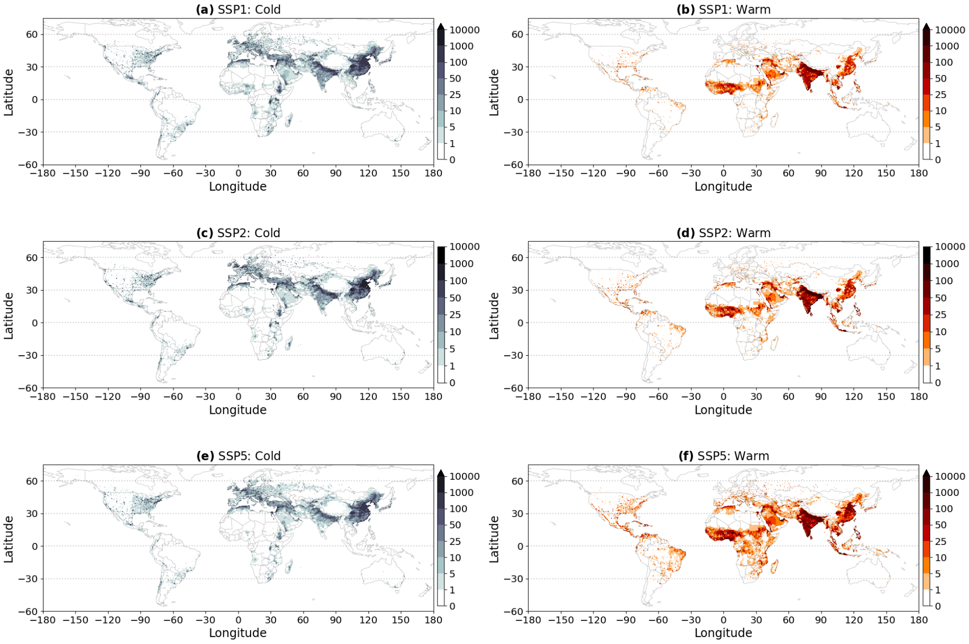

Supplementary Figure 9 Change in mortality across the century (2090-2000) due to exposure to cold (left) and warm (right) temperatures for the Shared Socio-economic Pathways SSP1-2.6 scenario (a and b), SSP2-4.5 scenario (c and d) and the SSP5-8.5 scenario (e and f).

Supplementary Table 1: Year 2000 mortality by Global Burden of Disease country and region. For each country and region, the population is shown in millions, along with the attributable mortality to non-optimal temperature and to air pollution, with their respective 95% confidence intervals shown in parenthesis.

**Country or Region**	**Pop.**	**Non-optimal temperature**	**air pollution**
Armenia	3.74	2,006 (871 - 3,237)	4,743 (2,971 - 6,988)
Azerbaijan	7.01	3,800 (1,504 - 6,471)	6,579 (3,893 - 10,283)
Georgia	5.23	3,955 (1,806 - 6,470)	6,998 (3,980 - 11,340)
Kazakhstan	16.17	7,682 (2,633 - 13,293)	12,109 (6,782 - 20,089)
Kyrgyzstan	5.33	1,915 (854 - 3,057)	3,300 (1,946 - 5,121)
Mongolia	2.5	1,090 (204 - 2,200)	2,088 (1,168 - 3,566)
Tajikistan	6.24	2,185 (984 - 3,643)	3,550 (2,046 - 5,678)
Turkmenistan	4.79	2,175 (759 - 3,650)	4,453 (2,931 - 6,369)
Uzbekistan	24.06	12,163 (4,711 - 20,017)	25,542 (16,716 - 36,709)
Albania	3.03	1,015 (511 - 1,581)	1,490 (837 - 2,434)
Bosnia and Herzegovina	3.96	2,172 (1,050 - 3,620)	4,955 (2,904 - 7,872)
Bulgaria	8.0	8,839 (4,125 - 14,143)	16,606 (10,263 - 24,874)
Croatia	4.2	2,777 (1,307 - 4,394)	4,356 (2,496 - 6,969)
Czech Republic	10.34	5,623 (3,192 - 8,334)	9,574 (5,481 - 15,277)
Hungary	10.0	6,418 (3,006 - 10,206)	12,066 (7,103 - 18,800)
Rep. of Macedonia	2.09	1,438 (724 - 2,256)	3,387 (2,237 - 4,841)
Montenegro	0.65	329 (171 - 527)	604 (345 - 972)
Poland	38.45	19,917 (11,131 - 28,888)	40,388 (25,236 - 59,303)
Romania	22.09	17,048 (8,150 - 26,518)	26,240 (15,337 - 41,230)
Serbia	9.77	7,415 (3,469 - 12,060)	14,921 (8,991 - 23,058)
Slovakia	5.39	2,657 (1,308 - 4,186)	4,937 (2,852 - 7,809)
Slovenia	2.29	1,118 (502 - 1,887)	1,442 (753 - 2,559)
Belarus	10.02	6,770 (3,570 - 10,162)	11,050 (5,974 - 18,392)
Estonia	1.3	880 (396 - 1,436)	582 (214 - 1,282)
Latvia	2.4	1,839 (982 - 2,778)	2,420 (1,318 - 4,049)
Lithuania	3.66	2,133 (1,122 - 3,216)	3,120 (1,637 - 5,319)
Rep. of Moldova	4.26	2,697 (1,215 - 4,245)	4,525 (2,547 - 7,270)
Russian Federation	143.0	109,014 (45,547 - 171,081)	146,334 (80,763 - 240,404)
Ukraine	49.04	40,897 (20,412 - 62,388)	72,103 (41,572 - 113,828)
Australia	17.58	4,797 (2,513 - 7,228)	1,483 (250 - 4,322)
New Zealand	3.56	1,214 (705 - 1,768)	341 (65 - 960)
Brunei Darussalam	0.27	4 (0 - 23)	33 (11 - 77)

Japan	120.46	57,448 (27,869 - 86,557)	35,841 (17,700 - 63,121)
Singapore	3.64	61 (0 - 326)	825 (451 - 1,358)
Republic of Korea	46.2	13,648 (4,934 - 23,561)	24,606 (14,352 - 38,210)
Canada	29.92	9,237 (3,569 - 15,247)	4,172 (1,259 - 9,943)
United States of America	277.12	101,810 (39,586 - 161,888)	72,742 (31,441 - 140,180)
Argentina	36.66	11,450 (5,660 - 18,062)	11,525 (5,865 - 20,258)
Chile	14.3	4,046 (2,369 - 5,866)	4,430 (2,535 - 7,042)
Uruguay	3.19	1,167 (619 - 1,785)	1,041 (482 - 1,973)
Andorra	0.0	0 (0 - 0)	0 (0 - 0)
Austria	8.19	3,669 (1,713 - 5,862)	4,234 (2,194 - 7,343)
Belgium	10.35	4,764 (2,714 - 7,011)	4,846 (2,460 - 8,493)
Cyprus	0.6	244 (30 - 501)	307 (159 - 530)
Denmark	4.25	1,960 (1,138 - 2,842)	1,500 (657 - 2,903)
Finland	5.01	2,376 (1,022 - 3,870)	769 (169 - 2,069)
France	57.6	19,205 (10,173 - 29,249)	16,832 (8,171 - 30,613)
Germany	81.8	39,216 (20,880 - 60,046)	43,038 (21,325 - 76,927)
Greece	9.55	5,315 (2,563 - 8,301)	5,585 (3,089 - 9,157)
Greenland	0.02	24 (12 - 38)	0 (0 - 0)
Iceland	0.22	74 (38 - 114)	9 (0 - 37)
Ireland	3.75	1,526 (937 - 2,156)	759 (281 - 1,622)
Israel	6.4	1,209 (188 - 2,324)	2,061 (1,155 - 3,294)
Italy	53.69	26,548 (12,265 - 41,319)	32,207 (18,574 - 50,857)
San Marino	0.0	0 (0 - 0)	0 (0 - 0)
Luxembourg	0.38	132 (71 - 202)	142 (69 - 261)
Malta	0.0	0 (0 - 0)	0 (0 - 0)
Netherlands	14.69	5,510 (3,125 - 8,109)	5,892 (2,944 - 10,386)
Norway	4.03	1,735 (892 - 2,588)	566 (157 - 1,389)
Portugal	9.29	5,355 (3,122 - 7,772)	3,334 (1,454 - 6,605)
Spain	36.23	15,649 (7,864 - 24,096)	10,530 (4,760 - 19,963)
Sweden	8.45	3,749 (1,853 - 5,858)	1,399 (398 - 3,476)
Switzerland	7.23	2,650 (1,357 - 4,064)	2,490 (1,188 - 4,543)
United Kingdom	57.67	29,710 (18,594 - 41,072)	22,218 (10,652 - 40,129)
Bolivia	8.32	1,636 (775 - 2,966)	3,054 (1,427 - 5,752)
Ecuador	12.18	1,301 (716 - 2,203)	2,325 (1,184 - 4,060)
Peru	22.24	3,075 (1,661 - 4,983)	4,851 (2,656 - 8,187)
Antigua and Barbuda	0.0	0 (0 - 0)	0 (0 - 0)
Bahamas	0.01	0 (0 - 1)	1 (0 - 2)
Barbados	0.0	0 (0 - 0)	0 (0 - 0)
Belize	0.22	5 (0 - 17)	40 (20 - 71)

Bermuda	0.0	0 (0 - 0)	0 (0 - 0)
Cuba	10.21	683 (0 - 1,760)	2,451 (1,044 - 4,856)
Dominica	0.0	0 (0 - 0)	0 (0 - 0)
Dominican Republic	7.45	282 (51 - 713)	1,343 (576 - 2,738)
Grenada	0.0	0 (0 - 0)	0 (0 - 0)
Guyana	0.67	12 (0 - 80)	327 (171 - 564)
Haiti	7.46	261 (0 - 1,119)	2,240 (831 - 5,098)
Jamaica	1.86	55 (0 - 187)	559 (285 - 972)
Puerto Rico	3.67	205 (0 - 565)	428 (92 - 1,124)
United States Virgin Islands	0.0	0 (0 - 0)	0 (0 - 0)
Saint Kitts and Nevis	0.0	0 (0 - 0)	0 (0 - 0)
Saint Lucia	0.0	0 (0 - 0)	0 (0 - 0)
Saint Vincent and Grenadines	0.0	0 (0 - 0)	0 (0 - 0)
Suriname	0.42	7 (0 - 39)	149 (78 - 258)
Trinidad and Tobago	1.07	20 (0 - 120)	511 (270 - 860)
Colombia	41.19	3,742 (1,940 - 6,172)	9,272 (4,985 - 15,365)
Costa Rica	3.97	164 (45 - 344)	780 (414 - 1,307)
El Salvador	6.12	156 (6 - 477)	1,986 (1,162 - 3,110)
Guatemala	11.34	1,243 (563 - 2,244)	3,506 (1,986 - 5,682)
Honduras	6.37	294 (70 - 739)	1,984 (957 - 3,687)
Mexico	97.62	14,142 (6,553 - 22,261)	24,338 (13,686 - 38,202)
Nicaragua	4.94	117 (19 - 383)	1,034 (547 - 1,749)
Panama	2.78	34 (4 - 146)	450 (222 - 807)
Venezuela	22.07	889 (152 - 2,145)	6,632 (3,773 - 10,544)
Brazil	164.77	14,077 (4,402 - 27,304)	42,360 (21,722 - 73,531)
Paraguay	5.26	352 (30 - 777)	939 (454 - 1,746)
Afghanistan	22.11	9,210 (3,132 - 18,643)	16,809 (8,066 - 31,528)
Algeria	29.53	7,498 (2,491 - 14,744)	8,721 (3,835 - 17,789)
Bahrain	0.1	17 (0 - 38)	0 (0 - 0)
Egypt	65.19	16,568 (671 - 38,088)	55,491 (34,097 - 85,850)
Iran	69.93	17,210 (5,005 - 29,996)	38,221 (25,268 - 54,237)
Iraq	22.87	5,568 (152 - 13,326)	13,416 (7,736 - 21,880)
Jordan	4.87	719 (158 - 1,454)	1,805 (1,004 - 2,956)
Kuwait	1.32	114 (13 - 228)	374 (265 - 506)
Lebanon	3.13	850 (342 - 1,528)	1,331 (697 - 2,279)
Libya	5.13	583 (28 - 1,410)	1,463 (763 - 2,680)
Morocco	28.12	7,309 (2,720 - 13,904)	12,246 (5,807 - 23,276)

State of Palestine	2.44	273 (48 - 572)	610 (315 - 1,072)
Oman	2.3	332 (22 - 747)	1,186 (719 - 1,844)
Qatar	0.33	31 (0 - 74)	111 (69 - 173)
Saudi Arabia	19.96	3,013 (133 - 6,911)	11,270 (7,106 - 16,807)
Sudan	24.89	4,404 (16 - 12,567)	15,762 (7,755 - 29,123)
Syrian Arab Republic	15.85	4,022 (928 - 7,785)	9,262 (5,498 - 14,611)
Tunisia	9.18	2,242 (437 - 4,712)	3,749 (1,784 - 7,062)
United Arab Emirates	2.55	189 (2 - 528)	723 (406 - 1,210)
Turkey	64.96	15,125 (5,891 - 27,948)	30,058 (15,980 - 51,531)
Yemen	17.86	2,441 (698 - 5,563)	8,842 (4,444 - 16,394)
Bangladesh	134.58	11,250 (50 - 30,293)	108,696 (67,578 - 165,524)
Bhutan	2.25	441 (168 - 874)	859 (397 - 1,666)
India	995.26	145,643 (3,312 - 342,220)	727,792 (481,665 - 1,038,765)
Nepal	23.08	4,181 (1,189 - 8,811)	13,566 (7,291 - 22,621)
Pakistan	143.45	33,856 (1,948 - 79,761)	113,389 (69,903 - 173,830)
South Asia	1,298.62	195,371 (6,667 - 461,959)	964,302 (626,833 - 1,402,407)
Angola	13.14	1,015 (225 - 2,585)	4,111 (1,738 - 8,724)
Central African Republic	3.48	141 (0 - 653)	2,295 (1,037 - 4,514)
Congo	3.11	89 (0 - 368)	1,433 (701 - 2,644)
Dem. Rep. of the Congo	51.13	1,937 (405 - 6,488)	21,322 (10,191 - 40,825)
Equatorial Guinea	0.32	7 (0 - 35)	122 (53 - 246)
Gabon	0.93	20 (0 - 105)	357 (165 - 678)
Burundi	6.49	648 (182 - 1,449)	2,884 (1,409 - 5,357)
Comoros	0.0	0 (0 - 0)	0 (0 - 0)
Djibouti	0.5	67 (0 - 213)	144 (68 - 273)
Eritrea	3.61	322 (42 - 971)	1,282 (576 - 2,515)
Ethiopia	63.03	10,742 (4,836 - 19,546)	19,608 (9,498 - 36,529)
Kenya	29.64	2,176 (906 - 4,145)	4,986 (2,444 - 9,158)
Madagascar	15.64	1,912 (659 - 3,879)	3,483 (1,454 - 7,301)
Malawi	11.03	733 (101 - 1,808)	2,962 (1,342 - 5,796)
Mozambique	18.13	877 (29 - 2,751)	4,238 (1,804 - 8,863)
Rwanda	7.45	1,179 (449 - 2,347)	3,621 (1,765 - 6,648)
Somalia	8.63	495 (2 - 2,095)	2,398 (894 - 5,509)
South Sudan	6.06	431 (0 - 1,537)	2,037 (867 - 4,183)
United Republic of Tanzania	33.49	1,654 (242 - 4,440)	8,518 (3,886 - 16,567)
Uganda	23.12	835 (113 - 2,427)	6,188 (2,851 - 11,804)

Zambia	10.42	820 (90 - 2,006)	3,385 (1,559 - 6,561)
Botswana	1.53	213 (11 - 583)	481 (187 - 1,069)
Lesotho	2.01	702 (357 - 1,213)	904 (435 - 1,681)
Namibia	1.71	192 (33 - 453)	464 (204 - 923)
South Africa	40.9	7,739 (3,859 - 12,380)	14,352 (8,475 - 22,616)
Swaziland	0.96	135 (38 - 295)	312 (130 - 643)
Zimbabwe	12.57	1,197 (310 - 2,605)	2,622 (1,161 - 5,281)
Benin	5.29	307 (0 - 1,137)	2,329 (1,193 - 4,096)
Burkina Faso	11.53	1,181 (0 - 3,644)	4,615 (2,292 - 8,279)
Cameroon	14.78	781 (44 - 2,581)	7,475 (3,960 - 12,939)
Cape Verde	0.0	0 (0 - 0)	0 (0 - 0)
Chad	7.9	974 (0 - 3,166)	4,031 (1,980 - 7,376)
Cote d'Ivoire	15.64	424 (0 - 2,152)	5,482 (2,468 - 10,713)
Gambia	1.28	37 (0 - 146)	369 (184 - 660)
Ghana	18.94	643 (0 - 2,540)	6,426 (3,324 - 11,282)
Guinea	8.08	289 (0 - 1,278)	3,462 (1,646 - 6,482)
Guinea-Bissau	1.13	53 (0 - 229)	560 (279 - 1,018)
Liberia	2.89	28 (0 - 239)	671 (285 - 1,396)
Mali	11.31	1,093 (0 - 3,403)	3,995 (1,977 - 7,217)
Mauritania	2.52	276 (0 - 795)	921 (490 - 1,575)
Niger	10.81	1,996 (0 - 6,308)	8,841 (4,636 - 15,555)
Nigeria	111.54	6,710 (2 - 24,263)	73,659 (40,597 - 125,342)
Sao Tome and Principe	0.0	0 (0 - 0)	0 (0 - 0)
Senegal	6.49	270 (0 - 944)	2,116 (1,091 - 3,672)
Sierra Leone	4.29	70 (0 - 485)	1,448 (611 - 2,927)
Togo	4.41	181 (0 - 698)	1,685 (857 - 3,017)
China	1,248.49	519,346 (213,257 - 843,738)	1,492,873 (1,002,264 - 2,061,741)
Dem. People's Rep. of Korea	21.37	10,263 (3,765 - 19,729)	19,306 (9,799 - 34,409)
Taiwan	18.27	2,378 (676 - 4,338)	5,866 (3,500 - 8,984)
Cambodia	13.08	744 (0 - 2,759)	4,702 (2,167 - 9,138)
Indonesia	189.1	3,035 (44 - 18,090)	80,390 (44,992 - 132,678)
Lao People's Dem. Rep.	5.41	459 (32 - 1,335)	3,318 (1,637 - 6,177)
Malaysia	21.09	283 (3 - 1,589)	5,455 (2,769 - 9,697)
Maldives	0.0	0 (0 - 0)	0 (0 - 0)
Mauritius	0.69	72 (11 - 145)	275 (143 - 473)
Myanmar	47.1	5,414 (351 - 16,705)	45,990 (24,350 - 80,035)
Philippines	64.79	1,113 (20 - 4,725)	14,349 (8,019 - 23,657)
Seychelles	0.0	0 (0 - 0)	0 (0 - 0)
Sri Lanka	14.8	717 (23 - 2,404)	7,212 (4,009 - 11,946)

Thailand	61.27	4,430 (8 - 12,551)	29,377 (15,743 - 49,876)
Timor-Leste	0.65	7 (0 - 49)	95 (33 - 227)
Viet Nam	75.88	6,449 (55 - 19,927)	40,331 (20,988 - 68,523)
American Samoa	0.0	0 (0 - 0)	0 (0 - 0)
Micronesia	0.0	0 (0 - 0)	0 (0 - 0)
Fiji	0.55	43 (0 - 113)	83 (12 - 256)
Guam	0.0	0 (0 - 0)	0 (0 - 0)
Kiribati	0.0	0 (0 - 0)	0 (0 - 0)
Nauru	0.0	0 (0 - 0)	0 (0 - 0)
Niue	0.0	0 (0 - 0)	0 (0 - 0)
Northern Mariana Islands	0.0	0 (0 - 0)	0 (0 - 0)
Palau	0.0	0 (0 - 0)	0 (0 - 0)
Marshall Islands	0.0	0 (0 - 0)	0 (0 - 0)
Papua New Guinea	4.45	207 (31 - 698)	1,216 (436 - 2,873)
Western Samoa	0.0	0 (0 - 0)	0 (0 - 0)
Solomon Islands	0.11	2 (0 - 19)	28 (8 - 77)
Tonga	0.0	0 (0 - 0)	0 (0 - 0)
Tokelau	0.0	0 (0 - 0)	0 (0 - 0)
Vanuatu	0.03	1 (0 - 4)	4 (1 - 13)
Central-Eastern Europe and Central Asia	409.02	277,967 (126,217 - 435,944)	450,458 (261,295 - 712,682)
High-Income	932.28	375,502 (178,372 - 579,706)	315,758 (154,267 - 568,041)
Latin America and Caribbean	442.21	42,752 (16,986 - 77,745)	111,560 (58,542 - 190,270)
North Africa and Middle East	412.62	97,718 (22,887 - 200,767)	231,451 (131,613 - 382,809)
South Asia	1,298.62	195,371 (6,667 - 461,959)	964,302 (626,833 - 1,402,407)
Sub-Saharan Africa	607.87	51,591 (12,934 - 131,386)	242,592 (122,767 - 440,452)
Southeast-East Asia and Oceania	1,787.12	554,963 (218,276 - 948,919)	1,750,872 (1,140,869 - 2,500,780)
Central Asia	75.06	36,970 (14,326 - 62,038)	69,360 (42,434 - 106,142)
Central Europe	120.26	76,766 (38,647 - 118,599)	140,964 (84,835 - 215,997)
Eastern Europe	213.69	164,231 (73,244 - 255,307)	240,134 (134,026 - 390,543)
Australasia	21.14	6,011 (3,217 - 8,996)	1,824 (315 - 5,282)
High-Income Asia Pacific	170.56	71,161 (32,802 - 110,467)	61,304 (32,513 - 102,766)

High-Income North America	307.04	111,047 (43,155 - 177,135)	76,914 (32,700 - 150,123)
Southern Latin America	54.15	16,663 (8,648 - 25,712)	16,996 (8,882 - 29,272)
Western Europe	379.39	170,620 (90,549 - 257,395)	158,719 (79,857 - 280,598)
Andean Latin America	42.73	6,012 (3,152 - 10,152)	10,230 (5,266 - 17,999)
Caribbean	33.04	1,531 (51 - 4,602)	8,049 (3,368 - 16,542)
Central Latin America	196.41	20,780 (9,352 - 34,910)	49,981 (27,732 - 80,453)
Tropical Latin America	170.03	14,429 (4,432 - 28,080)	43,299 (22,176 - 75,277)
North Africa and Middle East	412.62	97,718 (22,887 - 200,767)	231,451 (131,613 - 382,809)
Central Sub-Saharan Africa	72.11	3,209 (630 - 10,234)	29,640 (13,887 - 57,630)
Eastern Sub-Saharan Africa	237.25	22,890 (7,651 - 49,614)	65,733 (30,418 - 127,065)
Southern Sub-Saharan Africa	59.67	10,178 (4,607 - 17,530)	19,134 (10,592 - 32,213)
Western Sub-Saharan Africa	238.83	15,314 (46 - 54,008)	128,085 (67,871 - 223,544)
East Asia	1,288.13	531,987 (217,698 - 867,805)	1,518,045 (1,015,562 - 2,105,135)
Southeast Asia	493.85	22,722 (547 - 80,280)	231,495 (124,850 - 392,427)
Oceania	5.13	254 (31 - 834)	1,331 (457 - 3,218)
WORLD	5,871.45	1,593,486 (581,663 - 2,832,088)	4,061,127 (2,492,687 - 6,188,457)

Supplementary Table 2: Year 2090 mortality by Global Burden of Disease country and region for the Shared Socio-economic Pathways SSP1-2.6 scenario. For each country and region, the population is shown in millions, along with the attributable mortality to non-optimal temperature and to air pollution, with their respective 95% confidence intervals shown in parenthesis.

**Country or Region**	**Pop.**	**Non-optimal temperature**	**air pollution**
Armenia	1.59	7,404 (2,928 - 12,214)	9,095 (5,012 - 14,662)
Azerbaijan	6.44	29,722 (10,280 - 51,952)	29,028 (15,524 - 49,130)
Georgia	1.91	8,320 (3,111 - 14,253)	6,058 (2,825 - 11,511)
Kazakhstan	15.71	58,907 (19,186 - 102,006)	40,580 (20,246 - 76,003)
Kyrgyzstan	4.67	16,277 (6,690 - 26,514)	19,928 (10,601 - 32,839)
Mongolia	2.83	9,712 (2,187 - 19,372)	10,911 (5,529 - 20,299)
Tajikistan	5.49	18,014 (6,279 - 32,879)	24,086 (13,420 - 39,801)
Turkmenistan	4.59	19,110 (4,488 - 33,631)	30,533 (19,790 - 43,628)
Uzbekistan	22.76	142,900 (50,141 - 237,494)	206,686 (129,871 - 305,034)
Albania	2.1	8,895 (3,726 - 14,646)	3,806 (1,460 - 8,229)
Bosnia and Herzegovina	2.11	8,692 (3,506 - 15,646)	4,871 (1,913 - 10,634)
Bulgaria	4.51	27,939 (10,811 - 46,926)	11,876 (4,612 - 25,360)
Croatia	2.72	11,645 (4,398 - 19,781)	2,642 (649 - 7,162)
Czech Republic	11.61	29,791 (14,616 - 46,746)	1,953 (111 - 11,109)
Hungary	7.91	23,265 (9,099 - 38,848)	2,928 (209 - 11,322)
Rep. of Macedonia	1.81	12,165 (5,061 - 20,494)	8,852 (4,455 - 16,027)
Montenegro	0.39	1,637 (765 - 2,728)	708 (265 - 1,579)
Poland	29.77	79,732 (37,789 - 123,224)	7,671 (964 - 33,612)
Romania	10.41	55,389 (20,140 - 92,926)	8,803 (1,491 - 27,932)
Serbia	7.11	40,660 (15,753 - 70,005)	15,154 (5,348 - 35,053)
Slovakia	4.94	14,219 (6,414 - 22,943)	1,339 (14 - 6,323)
Slovenia	2.28	5,027 (1,935 - 8,988)	751 (117 - 2,549)
Belarus	5.49	19,874 (9,606 - 30,523)	261 (0 - 6,152)
Estonia	1.17	2,965 (1,487 - 4,595)	0 (0 - 62)
Latvia	1.23	4,788 (2,627 - 7,065)	144 (0 - 1,327)
Lithuania	1.73	5,687 (2,862 - 8,647)	22 (0 - 1,271)
Rep. of Moldova	1.06	5,736 (2,048 - 9,504)	639 (24 - 2,642)
Russian Federation	101.45	400,977 (173,552 - 621,688)	36,888 (8,455 - 161,569)
Ukraine	28.0	126,651 (49,923 - 205,323)	18,042 (2,013 - 66,813)
Australia	39.97	50,719 (22,440 - 80,998)	7,084 (680 - 27,876)
New Zealand	6.37	11,330 (6,307 - 16,796)	1,803 (320 - 5,586)

Brunei Darussalam	0.4	299 (0 - 1,118)	385 (67 - 1,140)
Japan	81.27	156,723 (65,803 - 247,483)	17,924 (3,927 - 53,928)
Singapore	4.93	2,544 (0 - 8,096)	3,443 (1,340 - 7,207)
Republic of Korea	35.44	111,837 (33,451 - 206,041)	36,581 (12,634 - 85,467)
Canada	54.77	85,225 (33,755 - 140,109)	34 (0 - 2,637)
United States of America	461.31	695,304 (222,000 - 1,164,598)	61,711 (18,825 - 198,024)
Argentina	37.01	80,550 (33,473 - 135,302)	44,580 (19,752 - 86,115)
Chile	13.79	44,674 (25,295 - 65,104)	19,461 (9,090 - 35,937)
Uruguay	2.07	4,727 (2,086 - 7,733)	2,764 (1,169 - 5,558)
Andorra	0.0	0 (0 - 0)	0 (0 - 0)
Austria	8.87	20,995 (8,610 - 35,109)	1,121 (38 - 6,653)
Belgium	13.87	26,063 (13,638 - 39,722)	608 (0 - 6,108)
Cyprus	1.03	4,796 (86 - 10,488)	3,159 (1,439 - 6,028)
Denmark	6.17	10,575 (5,805 - 15,858)	2 (0 - 1,720)
Finland	6.88	11,841 (4,905 - 19,681)	0 (0 - 58)
France	82.03	110,546 (53,627 - 173,676)	12,052 (1,922 - 43,490)
Germany	72.94	157,780 (74,029 - 253,740)	1,884 (15 - 28,702)
Greece	9.41	24,035 (8,621 - 40,739)	8,341 (3,327 - 17,466)
Greenland	0.02	62 (30 - 101)	0 (0 - 0)
Iceland	0.5	943 (465 - 1,474)	33 (0 - 235)
Ireland	7.26	17,560 (10,450 - 25,012)	529 (0 - 4,490)
Israel	20.24	16,392 (492 - 33,838)	15,111 (7,417 - 26,542)
Italy	50.94	105,935 (40,177 - 176,749)	33,111 (11,839 - 73,606)
San Marino	0.0	0 (0 - 0)	0 (0 - 0)
Luxembourg	0.81	1,569 (775 - 2,475)	35 (0 - 363)
Malta	0.0	0 (0 - 0)	0 (0 - 0)
Netherlands	17.77	32,588 (17,413 - 49,071)	386 (0 - 7,057)
Norway	8.24	13,917 (6,854 - 21,222)	3 (0 - 1,651)
Portugal	10.03	26,705 (13,951 - 40,408)	6,600 (1,822 - 16,797)
Spain	45.42	92,979 (40,015 - 150,880)	20,833 (5,919 - 52,756)
Sweden	14.67	22,465 (11,168 - 35,228)	0 (0 - 735)
Switzerland	9.35	16,600 (7,673 - 26,412)	1,399 (164 - 5,945)
United Kingdom	84.2	168,182 (100,626 - 237,731)	2,772 (82 - 36,322)
Bolivia	10.38	15,599 (6,677 - 30,288)	24,199 (11,435 - 45,605)
Ecuador	15.04	22,811 (11,675 - 39,789)	22,192 (10,281 - 41,060)
Peru	18.59	25,332 (12,156 - 43,046)	23,577 (11,551 - 43,252)
Antigua and Barbuda	0.0	0 (0 - 0)	0 (0 - 0)
Bahamas	0.01	4 (0 - 11)	3 (1 - 7)

Barbados	0.0	0 (0 - 0)	0 (0 - 0)
Belize	0.32	184 (0 - 520)	548 (259 - 1,001)
Bermuda	0.0	0 (0 - 0)	0 (0 - 0)
Cuba	4.51	4,038 (0 - 9,732)	5,231 (1,944 - 11,243)
Dominica	0.0	0 (0 - 0)	0 (0 - 0)
Dominican Republic	8.35	2,534 (164 - 7,717)	10,078 (4,236 - 20,684)
Grenada	0.0	0 (0 - 0)	0 (0 - 0)
Guyana	0.38	225 (0 - 847)	1,618 (865 - 2,723)
Haiti	9.29	3,918 (0 - 17,977)	20,517 (7,412 - 47,631)
Jamaica	1.25	259 (0 - 1,081)	2,540 (1,246 - 4,445)
Puerto Rico	1.55	505 (0 - 1,712)	1,068 (245 - 2,770)
United States Virgin Islands	0.0	0 (0 - 0)	0 (0 - 0)
Saint Kitts and Nevis	0.0	0 (0 - 0)	0 (0 - 0)
Saint Lucia	0.0	0 (0 - 0)	0 (0 - 0)
Saint Vincent and Grenadines	0.0	0 (0 - 0)	0 (0 - 0)
Suriname	0.49	195 (0 - 656)	1,123 (580 - 1,935)
Trinidad and Tobago	0.62	213 (0 - 902)	2,100 (1,085 - 3,547)
Colombia	48.42	50,797 (21,135 - 88,046)	86,481 (42,347 - 149,629)
Costa Rica	5.19	2,137 (463 - 5,430)	10,139 (5,039 - 17,372)
El Salvador	3.73	689 (2 - 2,626)	7,918 (4,194 - 12,997)
Guatemala	19.12	22,541 (7,774 - 43,445)	51,478 (28,785 - 83,840)
Honduras	8.84	5,322 (832 - 14,588)	27,846 (13,066 - 52,236)
Mexico	109.03	186,874 (75,518 - 304,436)	123,747 (50,672 - 241,959)
Nicaragua	4.63	2,126 (96 - 6,660)	10,147 (5,102 - 17,735)
Panama	3.96	921 (21 - 3,464)	5,479 (2,508 - 10,124)
Venezuela	30.53	17,501 (2,520 - 42,310)	69,558 (36,864 - 114,075)
Brazil	155.97	129,709 (25,845 - 273,260)	172,260 (65,484 - 357,657)
Paraguay	7.35	5,331 (120 - 12,799)	8,080 (3,307 - 16,730)
Afghanistan	67.36	127,507 (29,822 - 274,647)	248,997 (123,777 - 437,546)
Algeria	36.9	173,602 (42,264 - 349,360)	103,949 (44,166 - 218,260)
Bahrain	0.54	2,405 (0 - 5,517)	0 (0 - 0)
Egypt	100.21	263,387 (5,787 - 621,567)	532,161 (308,943 - 854,061)
Iran	66.2	264,475 (64,249 - 480,512)	351,940 (210,417 - 527,910)
Iraq	62.24	196,342 (6,548 - 456,128)	348,853 (205,019 - 549,119)
Jordan	12.54	31,299 (902 - 70,193)	48,452 (25,167 - 82,749)
Kuwait	3.3	6,917 (0 - 15,907)	12,263 (7,657 - 17,608)
Lebanon	3.24	9,981 (3,081 - 19,316)	7,893 (3,597 - 14,852)
Libya	7.69	13,079 (101 - 32,410)	17,448 (8,114 - 33,206)

Morocco	24.1	66,101 (17,703 - 139,952)	67,387 (29,455 - 135,089)
State of Palestine	3.54	8,061 (108 - 18,210)	9,558 (4,476 - 18,033)
Oman	3.38	11,434 (592 - 25,443)	22,424 (13,424 - 34,726)
Qatar	1.79	9,937 (0 - 23,564)	21,564 (13,159 - 33,072)
Saudi Arabia	49.11	129,169 (1,429 - 302,113)	319,134 (197,507 - 473,730)
Sudan	54.48	89,096 (680 - 232,803)	214,182 (110,666 - 369,886)
Syrian Arab Republic	27.16	122,301 (9,742 - 252,591)	154,041 (84,569 - 257,296)
Tunisia	9.09	28,562 (2,731 - 62,418)	24,238 (10,743 - 48,328)
United Arab Emirates	13.62	78,776 (30 - 212,837)	147,156 (86,467 - 239,167)
Turkey	70.27	185,516 (60,233 - 360,542)	136,441 (58,923 - 272,852)
Yemen	49.81	57,869 (14,175 - 128,797)	174,720 (92,015 - 301,477)
Bangladesh	136.93	236,696 (213 - 625,236)	984,876 (570,311 - 1,661,206)
Bhutan	0.99	1,848 (624 - 3,811)	2,943 (1,326 - 5,771)
India	1,258.81	2,216,421 (19,022 - 5,388,512)	6,249,867 (3,861,299 - 9,395,782)
Nepal	39.33	69,720 (10,293 - 171,970)	211,255 (109,166 - 353,508)
Pakistan	230.15	411,226 (9,600 - 1,042,759)	1,225,157 (734,496 - 1,922,608)
South Asia	1,666.21	2,935,911 (39,752 - 7,232,287)	8,674,098 (5,276,599 - 13,338,876)
Angola	42.89	18,983 (3,292 - 49,865)	79,383 (36,997 - 152,441)
Central African Republic	5.9	1,616 (0 - 8,340)	19,412 (9,011 - 37,249)
Congo	7.55	1,760 (0 - 9,217)	26,355 (13,051 - 48,059)
Dem. Rep. of the Congo	147.44	27,783 (4,190 - 118,233)	340,568 (170,270 - 628,682)
Equatorial Guinea	1.05	109 (0 - 744)	2,485 (1,156 - 4,788)
Gabon	1.44	501 (0 - 2,415)	4,529 (2,122 - 8,460)
Burundi	16.48	10,857 (2,343 - 25,766)	46,659 (23,268 - 84,435)
Comoros	0.0	0 (0 - 0)	0 (0 - 0)
Djibouti	0.94	1,433 (0 - 4,248)	1,944 (970 - 3,498)
Eritrea	10.59	9,896 (758 - 31,724)	26,097 (11,803 - 50,344)
Ethiopia	144.13	154,042 (56,098 - 310,840)	246,219 (116,965 - 460,698)
Kenya	72.2	42,364 (14,829 - 86,904)	89,845 (44,041 - 163,750)
Madagascar	38.01	36,413 (10,832 - 77,836)	58,210 (23,868 - 122,467)
Malawi	41.71	14,405 (1,239 - 38,281)	54,703 (26,057 - 102,326)
Mozambique	38.37	14,476 (242 - 45,932)	54,435 (24,644 - 106,129)
Rwanda	21.82	17,754 (5,488 - 38,657)	54,560 (26,711 - 99,318)
Somalia	11.79	4,990 (7 - 16,551)	12,399 (4,961 - 27,425)

South Sudan	12.64	9,422 (0 - 27,013)	20,994 (9,587 - 40,095)
United Republic of Tanzania	90.12	30,566 (3,362 - 87,109)	135,457 (64,236 - 253,245)
Uganda	97.12	17,631 (1,902 - 59,903)	153,187 (73,803 - 281,382)
Zambia	30.01	11,047 (498 - 29,426)	47,779 (23,599 - 87,020)
Botswana	2.3	2,620 (13 - 7,519)	4,875 (1,916 - 10,705)
Lesotho	2.14	4,461 (2,115 - 8,020)	4,274 (1,863 - 8,618)
Namibia	2.93	3,546 (295 - 9,402)	7,633 (3,442 - 15,010)
South Africa	50.2	69,330 (28,128 - 116,295)	98,733 (54,073 - 162,186)
Swaziland	1.38	1,208 (263 - 2,800)	2,686 (1,106 - 5,634)
Zimbabwe	9.8	7,996 (1,312 - 18,964)	14,821 (6,416 - 30,404)
Benin	15.87	13,652 (0 - 36,976)	46,433 (25,588 - 77,092)
Burkina Faso	37.53	40,661 (0 - 106,434)	81,730 (44,927 - 135,595)
Cameroon	29.89	14,044 (335 - 45,182)	99,474 (55,247 - 163,976)
Cape Verde	0.0	0 (0 - 0)	0 (0 - 0)
Chad	24.77	20,571 (0 - 59,214)	53,774 (28,697 - 92,859)
Cote d'Ivoire	25.08	15,251 (0 - 48,624)	60,115 (29,027 - 111,077)
Gambia	2.85	2,187 (0 - 6,374)	8,849 (4,869 - 14,693)
Ghana	40.13	34,400 (0 - 95,129)	118,813 (63,653 - 199,789)
Guinea	12.63	5,312 (0 - 18,774)	28,828 (14,700 - 50,854)
Guinea-Bissau	2.05	1,453 (0 - 4,383)	5,884 (3,131 - 10,119)
Liberia	10.8	2,143 (0 - 10,433)	20,010 (9,424 - 37,686)
Mali	33.97	33,769 (0 - 91,263)	82,329 (44,345 - 137,602)
Mauritania	5.06	7,093 (0 - 18,906)	14,884 (8,467 - 24,080)
Niger	51.41	39,275 (0 - 107,825)	99,153 (55,135 - 164,462)
Nigeria	418.23	187,301 (17 - 518,854)	901,964 (525,207 - 1,459,212)
Sao Tome and Principe	0.0	0 (0 - 0)	0 (0 - 0)
Senegal	12.47	10,092 (6 - 26,837)	34,966 (19,770 - 56,767)
Sierra Leone	10.33	3,532 (0 - 12,511)	20,122 (9,632 - 37,342)
Togo	9.34	8,452 (0 - 23,381)	29,085 (15,558 - 49,519)
China	761.53	4,453,514 (1,499,405 - 7,605,600)	4,802,908 (2,643,095 - 7,695,420)
Dem. People's Rep. of Korea	15.8	81,514 (25,074 - 166,470)	34,872 (12,200 - 85,147)
Taiwan	12.55	18,806 (2,602 - 39,251)	16,592 (7,245 - 32,006)
Cambodia	11.97	15,281 (0 - 40,284)	18,081 (7,183 - 39,859)
Indonesia	183.21	82,874 (195 - 274,005)	370,418 (175,288 - 703,670)
Lao People's Dem. Rep.	6.06	6,315 (29 - 18,392)	14,608 (6,300 - 30,431)
Malaysia	34.94	13,083 (127 - 49,654)	51,186 (21,234 - 106,446)
Maldives	0.0	0 (0 - 0)	0 (0 - 0)
Mauritius	0.66	488 (0 - 1,138)	1,464 (712 - 2,624)

Myanmar	29.43	38,085 (595 - 105,619)	124,680 (61,949 - 226,074)
Philippines	109.34	51,079 (120 - 159,641)	196,433 (104,457 - 329,680)
Seychelles	0.0	0 (0 - 0)	0 (0 - 0)
Sri Lanka	12.72	9,624 (151 - 27,256)	30,214 (14,243 - 56,294)
Thailand	48.05	56,701 (100 - 140,607)	71,715 (30,598 - 145,968)
Timor-Leste	1.36	64 (0 - 519)	856 (308 - 2,017)
Viet Nam	69.55	115,501 (888 - 310,135)	185,843 (75,026 - 381,999)
American Samoa	0.0	0 (0 - 0)	0 (0 - 0)
Micronesia	0.0	0 (0 - 0)	0 (0 - 0)
Fiji	0.39	221 (0 - 675)	417 (64 - 1,292)
Guam	0.0	0 (0 - 0)	0 (0 - 0)
Kiribati	0.0	0 (0 - 0)	0 (0 - 0)
Nauru	0.0	0 (0 - 0)	0 (0 - 0)
Niue	0.0	0 (0 - 0)	0 (0 - 0)
Northern Mariana Islands	0.0	0 (0 - 0)	0 (0 - 0)
Palau	0.0	0 (0 - 0)	0 (0 - 0)
Marshall Islands	0.0	0 (0 - 0)	0 (0 - 0)
Papua New Guinea	8.5	3,175 (387 - 11,278)	13,998 (4,829 - 33,570)
Western Samoa	0.0	0 (0 - 0)	0 (0 - 0)
Solomon Islands	0.17	29 (0 - 234)	281 (89 - 717)
Tonga	0.0	0 (0 - 0)	0 (0 - 0)
Tokelau	0.0	0 (0 - 0)	0 (0 - 0)
Vanuatu	0.05	16 (0 - 69)	66 (19 - 181)
Central-Eastern Europe and Central Asia	293.8	1,196,101 (481,407 - 1,941,561)	504,258 (254,918 - 1,029,634)
High-Income	1,208.0	2,126,459 (864,023 - 3,462,991)	303,749 (101,788 - 846,197)
Latin America and Caribbean	467.54	499,765 (164,997 - 951,340)	687,927 (308,508 - 1,300,259)
North Africa and Middle East	666.56	1,875,814 (260,178 - 4,084,826)	2,962,800 (1,638,262 - 4,918,969)
South Asia	1,666.21	2,935,911 (39,752 - 7,232,287)	8,674,098 (5,276,599 - 13,338,876)
Sub-Saharan Africa	1,643.37	954,397 (137,564 - 2,463,104)	3,314,653 (1,733,312 - 5,817,090)
Southeast-East Asia and Oceania	1,306.28	4,946,369 (1,529,673 - 8,950,826)	5,934,631 (3,164,837 - 9,873,397)
Central Asia	65.98	310,366 (105,288 - 530,315)	376,907 (222,818 - 592,908)

Central Europe	87.68	319,058 (134,014 - 523,902)	71,354 (21,609 - 196,890)
Eastern Europe	140.13	566,677 (242,105 - 887,344)	55,996 (10,492 - 239,836)
Australasia	46.34	62,049 (28,747 - 97,794)	8,888 (1,000 - 33,462)
High-Income Asia Pacific	122.04	271,402 (99,254 - 462,739)	58,332 (17,968 - 147,742)
High-Income North America	516.08	780,530 (255,754 - 1,304,707)	61,746 (18,825 - 200,661)
Southern Latin America	52.87	129,951 (60,855 - 208,138)	66,805 (30,012 - 127,610)
Western Europe	470.67	882,527 (419,412 - 1,389,613)	107,978 (33,984 - 336,723)
Andean Latin America	44.01	63,742 (30,509 - 113,123)	69,968 (33,267 - 129,918)
Caribbean	26.75	12,075 (164 - 41,153)	44,826 (17,873 - 95,987)
Central Latin America	233.46	288,908 (108,359 - 511,005)	392,793 (188,578 - 699,968)
Tropical Latin America	163.32	135,040 (25,965 - 286,059)	180,340 (68,791 - 374,386)
North Africa and Middle East	666.56	1,875,814 (260,178 - 4,084,826)	2,962,800 (1,638,262 - 4,918,969)
Central Sub-Saharan Africa	206.27	50,752 (7,481 - 188,814)	472,732 (232,607 - 879,678)
Eastern Sub-Saharan Africa	625.93	375,296 (97,598 - 880,188)	1,002,486 (474,515 - 1,882,130)
Southern Sub-Saharan Africa	68.76	89,160 (32,127 - 163,000)	133,022 (68,816 - 232,557)
Western Sub-Saharan Africa	742.41	439,189 (358 - 1,231,101)	1,706,413 (957,374 - 2,822,724)
East Asia	789.88	4,553,834 (1,527,081 - 7,811,321)	4,854,372 (2,662,541 - 7,812,574)
Southeast Asia	507.29	389,095 (2,205 - 1,127,250)	1,065,497 (497,296 - 2,025,063)
Oceania	9.1	3,440 (387 - 12,255)	14,762 (5,000 - 35,760)
WORLD	7,239.2	14,516,012 (3,474,991 - 29,047,685)	22,365,524 (12,470,979 - 37,092,415)

Supplementary Table 3: Year 2090 mortality by Global Burden of Disease country and region for the Shared Socio-economic Pathways SSP2-4.5 scenario. For each country and region, the population is shown in millions, along with the attributable mortality to non-optimal temperature and to air pollution, with their respective 95% confidence intervals shown in parenthesis.

**Country or Region**	**Pop.**	**Non-optimal temperature**	**air pollution**
Armenia	1.93	5,423 (1,960 - 9,088)	7,485 (4,291 - 11,717)
Azerbaijan	8.17	21,656 (6,611 - 38,378)	23,684 (12,925 - 39,473)
Georgia	2.27	6,129 (2,177 - 10,588)	5,653 (2,814 - 10,205)
Kazakhstan	19.46	45,039 (15,988 - 76,169)	38,095 (19,989 - 67,549)
Kyrgyzstan	6.15	12,306 (5,484 - 19,489)	17,598 (9,903 - 27,877)
Mongolia	3.71	6,540 (1,503 - 13,149)	10,115 (5,439 - 18,038)
Tajikistan	7.5	13,485 (4,051 - 25,020)	22,249 (13,176 - 34,942)
Turkmenistan	5.89	13,940 (2,203 - 25,296)	25,509 (17,039 - 35,797)
Uzbekistan	29.02	104,440 (31,980 - 177,081)	177,319 (116,391 - 253,340)
Albania	2.46	6,524 (2,244 - 11,277)	3,671 (1,555 - 7,436)
Bosnia and Herzegovina	2.41	6,599 (2,592 - 11,878)	5,177 (2,312 - 10,271)
Bulgaria	5.38	20,406 (7,942 - 34,138)	11,585 (5,095 - 22,858)
Croatia	3.23	9,093 (3,466 - 15,373)	3,451 (1,239 - 7,775)
Czech Republic	11.15	24,019 (11,251 - 38,153)	6,665 (1,468 - 17,956)
Hungary	7.34	16,571 (5,877 - 28,213)	5,132 (1,338 - 13,003)
Rep. of Macedonia	2.15	8,848 (3,485 - 15,025)	8,105 (4,351 - 13,848)
Montenegro	0.48	1,261 (547 - 2,152)	778 (332 - 1,588)
Poland	27.71	61,000 (28,326 - 94,657)	18,429 (5,044 - 45,833)
Romania	12.04	40,569 (14,992 - 67,585)	11,161 (3,018 - 28,139)
Serbia	8.35	29,595 (11,228 - 50,986)	16,474 (7,025 - 33,878)
Slovakia	4.66	10,826 (4,730 - 17,583)	3,228 (784 - 8,386)
Slovenia	2.23	4,094 (1,511 - 7,404)	1,144 (340 - 2,993)
Belarus	6.53	14,395 (6,547 - 22,544)	1,885 (35 - 8,346)
Estonia	1.1	2,156 (1,124 - 3,288)	5 (0 - 349)
Latvia	1.43	3,209 (1,662 - 4,859)	455 (38 - 1,707)
Lithuania	2.06	4,039 (1,923 - 6,259)	298 (4 - 1,853)
Rep. of Moldova	1.25	3,850 (1,125 - 6,588)	867 (116 - 2,555)
Russian Federation	126.04	287,187 (126,348 - 442,572)	56,038 (12,843 - 176,299)
Ukraine	34.15	89,106 (32,860 - 146,039)	25,516 (5,660 - 68,164)
Australia	40.3	42,427 (16,896 - 69,969)	7,609 (845 - 27,527)
New Zealand	6.29	9,290 (5,128 - 13,826)	1,885 (361 - 5,385)

Brunei Darussalam	0.57	571 (13 - 1,429)	497 (122 - 1,315)
Japan	77.85	133,648 (47,304 - 220,447)	25,559 (7,109 - 63,203)
Singapore	5.8	3,961 (0 - 9,331)	3,938 (1,728 - 7,617)
Republic of Korea	32.66	89,879 (28,916 - 162,501)	42,069 (16,646 - 88,304)
Canada	53.74	75,433 (29,929 - 123,744)	155 (7 - 7,734)
United States of America	447.31	546,955 (162,910 - 925,547)	74,235 (23,820 - 223,810)
Argentina	47.4	56,042 (19,043 - 99,127)	37,817 (17,134 - 72,253)
Chile	17.1	36,026 (20,433 - 52,507)	19,457 (9,585 - 34,526)
Uruguay	2.62	3,288 (1,206 - 5,675)	2,391 (1,030 - 4,754)
Andorra	0.0	0 (0 - 0)	0 (0 - 0)
Austria	8.61	17,870 (6,775 - 30,462)	3,604 (659 - 10,348)
Belgium	13.32	22,575 (11,591 - 34,632)	3,593 (438 - 11,032)
Cyprus	1.26	3,991 (12 - 8,778)	2,615 (1,186 - 5,013)
Denmark	5.88	8,089 (4,290 - 12,290)	551 (0 - 3,211)
Finland	6.63	10,370 (4,877 - 16,536)	23 (0 - 1,357)
France	81.12	98,349 (46,151 - 156,266)	22,416 (5,694 - 57,842)
Germany	70.2	138,039 (62,660 - 223,943)	17,214 (622 - 65,196)
Greece	9.05	20,031 (5,131 - 35,842)	8,278 (3,505 - 16,668)
Greenland	0.02	51 (26 - 82)	0 (0 - 0)
Iceland	0.49	773 (377 - 1,217)	44 (0 - 238)
Ireland	6.97	14,066 (8,285 - 20,148)	1,399 (5 - 5,375)
Israel	21.34	14,482 (77 - 30,504)	14,107 (7,074 - 24,417)
Italy	50.71	92,769 (31,812 - 158,087)	40,350 (16,843 - 81,050)
San Marino	0.0	0 (0 - 0)	0 (0 - 0)
Luxembourg	0.8	1,365 (656 - 2,172)	189 (6 - 673)
Malta	0.0	0 (0 - 0)	0 (0 - 0)
Netherlands	17.33	28,031 (14,846 - 42,338)	3,825 (167 - 13,007)
Norway	8.0	11,894 (6,216 - 17,734)	288 (0 - 2,914)
Portugal	9.86	19,957 (9,297 - 31,522)	8,498 (3,200 - 18,384)
Spain	45.14	80,637 (29,039 - 136,861)	28,318 (10,227 - 61,516)
Sweden	14.32	18,991 (9,563 - 29,633)	103 (0 - 3,611)
Switzerland	9.34	15,344 (6,954 - 24,541)	2,968 (531 - 8,566)
United Kingdom	82.4	137,296 (81,520 - 194,798)	16,268 (1,379 - 55,325)
Bolivia	14.64	11,101 (4,341 - 22,147)	22,440 (11,171 - 40,367)
Ecuador	19.43	17,009 (8,067 - 30,103)	23,213 (11,820 - 39,751)
Peru	23.72	16,737 (7,655 - 29,081)	23,985 (12,851 - 40,931)
Antigua and Barbuda	0.0	0 (0 - 0)	0 (0 - 0)
Bahamas	0.01	4 (0 - 11)	3 (1 - 7)

Barbados	0.0	0 (0 - 0)	0 (0 - 0)
Belize	0.44	217 (0 - 540)	510 (253 - 901)
Bermuda	0.0	0 (0 - 0)	0 (0 - 0)
Cuba	5.18	4,710 (47 - 10,449)	4,767 (1,862 - 9,950)
Dominica	0.0	0 (0 - 0)	0 (0 - 0)
Dominican Republic	11.08	2,493 (94 - 7,273)	9,010 (3,887 - 18,171)
Grenada	0.0	0 (0 - 0)	0 (0 - 0)
Guyana	0.5	324 (2 - 826)	1,381 (763 - 2,266)
Haiti	10.8	3,243 (2 - 11,848)	12,897 (4,839 - 28,863)
Jamaica	1.68	255 (0 - 970)	2,156 (1,087 - 3,718)
Puerto Rico	1.89	505 (0 - 1,715)	1,024 (263 - 2,552)
United States Virgin Islands	0.0	0 (0 - 0)	0 (0 - 0)
Saint Kitts and Nevis	0.0	0 (0 - 0)	0 (0 - 0)
Saint Lucia	0.0	0 (0 - 0)	0 (0 - 0)
Saint Vincent and Grenadines	0.0	0 (0 - 0)	0 (0 - 0)
Suriname	0.65	282 (5 - 667)	1,029 (554 - 1,717)
Trinidad and Tobago	0.72	313 (2 - 946)	1,792 (960 - 2,937)
Colombia	61.96	41,008 (15,769 - 71,772)	83,717 (43,924 - 137,628)
Costa Rica	6.68	1,511 (278 - 4,362)	9,248 (4,797 - 15,365)
El Salvador	4.95	773 (19 - 2,649)	7,222 (4,018 - 11,498)
Guatemala	28.54	13,983 (3,485 - 29,189)	43,378 (25,065 - 68,974)
Honduras	12.18	3,724 (256 - 11,017)	24,145 (11,704 - 44,325)
Mexico	144.52	139,563 (48,907 - 236,139)	164,368 (82,667 - 277,094)
Nicaragua	6.29	2,558 (51 - 6,433)	9,025 (4,742 - 15,211)
Panama	5.11	1,179 (12 - 3,403)	5,169 (2,511 - 9,131)
Venezuela	39.12	18,586 (1,748 - 42,765)	65,244 (36,305 - 103,538)
Brazil	193.0	107,620 (13,816 - 230,504)	188,090 (82,858 - 356,595)
Paraguay	9.78	3,940 (2 - 9,863)	7,874 (3,520 - 15,342)
Afghanistan	109.0	109,554 (22,447 - 242,427)	253,891 (129,978 - 441,472)
Algeria	45.96	113,782 (16,321 - 242,697)	87,428 (38,558 - 177,899)
Bahrain	0.71	1,882 (0 - 4,296)	0 (0 - 0)
Egypt	129.11	194,206 (2,250 - 459,726)	421,748 (245,924 - 675,204)
Iran	79.02	208,555 (47,089 - 379,089)	308,721 (191,730 - 450,666)
Iraq	86.8	127,612 (8,691 - 294,326)	307,593 (182,669 - 483,617)
Jordan	16.05	24,488 (13 - 55,548)	40,420 (21,423 - 68,171)
Kuwait	4.28	5,415 (0 - 12,386)	10,518 (6,844 - 14,789)
Lebanon	3.9	6,644 (1,650 - 13,340)	6,238 (2,931 - 11,489)
Libya	9.79	10,203 (41 - 25,656)	15,273 (7,254 - 28,885)

Morocco	30.89	43,374 (7,731 - 95,858)	64,358 (30,316 - 121,530)
State of Palestine	4.79	6,102 (0 - 13,896)	8,116 (3,894 - 15,033)
Oman	5.09	10,986 (302 - 24,701)	22,821 (13,968 - 34,848)
Qatar	2.27	8,256 (0 - 19,488)	18,868 (11,675 - 28,780)
Saudi Arabia	62.75	105,974 (648 - 249,401)	270,076 (170,258 - 397,060)
Sudan	74.98	70,998 (417 - 186,289)	173,471 (89,702 - 301,337)
Syrian Arab Republic	35.99	94,286 (3,143 - 197,866)	131,005 (73,140 - 216,362)
Tunisia	10.8	19,575 (1,022 - 43,532)	19,328 (8,741 - 37,913)
United Arab Emirates	17.01	64,936 (0 - 174,920)	127,896 (76,262 - 205,706)
Turkey	90.31	127,216 (38,638 - 248,862)	125,963 (58,071 - 239,245)
Yemen	74.09	41,437 (7,576 - 97,373)	159,955 (85,889 - 273,318)
Bangladesh	173.66	187,702 (195 - 481,042)	828,973 (502,580 - 1,333,447)
Bhutan	1.39	1,451 (487 - 3,101)	3,453 (1,711 - 6,267)
India	1,666.36	1,791,020 (14,355 - 4,296,730)	5,924,875 (3,887,553 - 8,485,542)
Nepal	52.72	52,098 (7,415 - 129,471)	198,050 (108,389 - 319,646)
Pakistan	327.68	325,661 (6,221 - 830,132)	1,035,562 (637,700 - 1,591,996)
South Asia	2,221.8	2,357,932 (28,673 - 5,740,477)	7,990,913 (5,137,933 - 11,736,899)
Angola	56.6	10,008 (849 - 29,396)	48,854 (21,950 - 96,780)
Central African Republic	7.63	1,605 (0 - 6,735)	13,810 (6,529 - 26,008)
Congo	9.4	1,420 (0 - 6,164)	15,972 (7,948 - 28,927)
Dem. Rep. of the Congo	186.67	18,541 (1,431 - 80,073)	227,050 (114,391 - 416,510)
Equatorial Guinea	1.32	75 (0 - 505)	1,603 (751 - 3,060)
Gabon	1.72	423 (0 - 1,558)	2,739 (1,307 - 5,064)
Burundi	20.12	4,530 (613 - 12,176)	30,705 (15,454 - 55,386)
Comoros	0.0	0 (0 - 0)	0 (0 - 0)
Djibouti	1.13	707 (0 - 2,054)	1,152 (584 - 2,064)
Eritrea	14.19	6,409 (220 - 20,532)	19,809 (9,321 - 37,153)
Ethiopia	191.16	75,883 (23,316 - 159,230)	181,927 (90,063 - 330,310)
Kenya	92.49	20,220 (5,949 - 43,830)	61,760 (31,541 - 108,666)
Madagascar	57.52	23,971 (5,594 - 55,921)	52,803 (22,061 - 109,775)
Malawi	61.81	8,469 (73 - 25,563)	34,757 (15,854 - 67,707)
Mozambique	50.38	10,241 (14 - 32,184)	31,586 (13,832 - 63,796)
Rwanda	29.48	7,808 (1,846 - 18,275)	38,547 (19,357 - 69,056)
Somalia	18.64	6,552 (1 - 19,459)	12,919 (5,256 - 28,050)

South Sudan	17.99	9,197 (0 - 25,308)	18,193 (8,305 - 34,866)
United Republic of Tanzania	133.31	14,751 (798 - 48,865)	97,411 (46,934 - 180,509)
Uganda	145.93	7,425 (546 - 32,431)	111,022 (54,609 - 201,339)
Zambia	42.06	6,166 (21 - 18,922)	29,464 (13,827 - 56,117)
Botswana	2.87	1,577 (0 - 4,837)	2,735 (1,014 - 6,320)
Lesotho	2.53	2,128 (974 - 3,878)	2,042 (846 - 4,308)
Namibia	3.44	1,810 (65 - 4,969)	4,319 (1,927 - 8,527)
South Africa	57.49	27,141 (7,975 - 49,660)	41,852 (21,181 - 73,668)
Swaziland	1.6	513 (76 - 1,328)	1,104 (399 - 2,561)
Zimbabwe	11.81	3,886 (184 - 10,204)	7,484 (3,061 - 16,102)
Benin	21.06	11,676 (0 - 29,830)	30,387 (16,799 - 50,440)
Burkina Faso	53.34	27,776 (0 - 71,625)	53,666 (29,240 - 89,778)
Cameroon	36.65	8,523 (9 - 27,255)	58,699 (32,425 - 97,623)
Cape Verde	0.0	0 (0 - 0)	0 (0 - 0)
Chad	33.39	14,676 (0 - 41,034)	35,476 (18,668 - 62,063)
Cote d'Ivoire	32.65	13,219 (2 - 36,378)	38,326 (18,534 - 70,804)
Gambia	3.86	1,562 (4 - 4,198)	4,913 (2,648 - 8,302)
Ghana	55.94	29,739 (4 - 73,936)	75,646 (41,113 - 126,575)
Guinea	16.66	4,031 (0 - 12,754)	17,990 (9,188 - 31,784)
Guinea-Bissau	2.64	1,286 (0 - 3,471)	4,003 (2,107 - 6,952)
Liberia	14.5	2,304 (0 - 7,834)	11,965 (5,625 - 22,642)
Mali	47.13	21,355 (0 - 57,280)	50,852 (27,413 - 85,352)
Mauritania	7.09	5,634 (0 - 14,956)	11,830 (6,744 - 19,135)
Niger	90.46	41,115 (0 - 113,107)	100,567 (55,266 - 168,931)
Nigeria	546.72	155,166 (0 - 408,799)	611,583 (350,571 - 1,008,029)
Sao Tome and Principe	0.0	0 (0 - 0)	0 (0 - 0)
Senegal	21.98	10,539 (25 - 27,164)	29,906 (16,692 - 49,070)
Sierra Leone	12.99	2,697 (0 - 8,121)	11,523 (5,396 - 21,687)
Togo	11.39	6,679 (0 - 17,071)	17,211 (9,267 - 29,269)
China	852.94	2,904,462 (830,537 - 5,114,478)	4,117,378 (2,392,170 - 6,305,538)
Dem. People's Rep. of Korea	18.55	53,707 (14,948 - 110,554)	33,982 (13,463 - 75,095)
Taiwan	12.55	11,107 (1,109 - 23,291)	12,943 (6,163 - 23,509)
Cambodia	16.06	14,557 (2 - 35,454)	15,999 (6,734 - 33,689)
Indonesia	217.11	117,630 (16,116 - 279,457)	331,812 (166,183 - 601,015)
Lao People's Dem. Rep.	8.29	4,901 (8 - 13,815)	13,059 (5,921 - 25,994)
Malaysia	43.31	18,977 (1,222 - 51,765)	50,427 (22,731 - 98,516)
Maldives	0.0	0 (0 - 0)	0 (0 - 0)
Mauritius	0.81	320 (0 - 841)	1,275 (638 - 2,239)

Myanmar	37.87	31,553 (270 - 82,760)	103,451 (53,560 - 182,088)
Philippines	142.03	57,109 (318 - 143,683)	160,682 (87,699 - 265,307)
Seychelles	0.0	0 (0 - 0)	0 (0 - 0)
Sri Lanka	17.8	11,698 (456 - 28,724)	29,210 (14,402 - 52,598)
Thailand	57.84	45,776 (362 - 108,812)	60,782 (27,754 - 117,501)
Timor-Leste	2.12	68 (0 - 469)	747 (267 - 1,756)
Viet Nam	82.13	114,449 (3,559 - 295,614)	176,503 (76,204 - 344,135)
American Samoa	0.0	0 (0 - 0)	0 (0 - 0)
Micronesia	0.0	0 (0 - 0)	0 (0 - 0)
Fiji	0.58	157 (0 - 543)	447 (88 - 1,287)
Guam	0.0	0 (0 - 0)	0 (0 - 0)
Kiribati	0.0	0 (0 - 0)	0 (0 - 0)
Nauru	0.0	0 (0 - 0)	0 (0 - 0)
Niue	0.0	0 (0 - 0)	0 (0 - 0)
Northern Mariana Islands	0.0	0 (0 - 0)	0 (0 - 0)
Palau	0.0	0 (0 - 0)	0 (0 - 0)
Marshall Islands	0.0	0 (0 - 0)	0 (0 - 0)
Papua New Guinea	11.34	2,625 (134 - 8,904)	12,845 (4,699 - 29,624)
Western Samoa	0.0	0 (0 - 0)	0 (0 - 0)
Solomon Islands	0.25	45 (0 - 246)	292 (97 - 729)
Tonga	0.0	0 (0 - 0)	0 (0 - 0)
Tokelau	0.0	0 (0 - 0)	0 (0 - 0)
Vanuatu	0.07	9 (0 - 54)	59 (17 - 160)
Central-Eastern Europe and Central Asia	346.23	872,305 (341,740 - 1,420,829)	507,770 (254,567 - 972,174)
High-Income	1,194.44	1,752,490 (671,933 - 2,892,488)	390,267 (129,924 - 982,169)
Latin America and Caribbean	602.86	391,639 (104,557 - 764,673)	711,688 (352,422 - 1,246,831)
North Africa and Middle East	893.58	1,395,481 (157,981 - 3,081,677)	2,573,685 (1,449,226 - 4,223,324)
South Asia	2,221.8	2,357,932 (28,673 - 5,740,477)	7,990,913 (5,137,933 - 11,736,899)
Sub-Saharan Africa	2,227.73	629,436 (50,587 - 1,668,872)	2,256,160 (1,175,999 - 3,981,063)
Southeast-East Asia and Oceania	1,521.64	3,389,151 (869,040 - 6,299,464)	5,121,896 (2,878,789 - 8,160,779)
Central Asia	84.1	228,959 (71,956 - 394,256)	327,707 (201,968 - 498,936)

Central Europe	89.58	239,405 (98,193 - 394,424)	94,999 (33,903 - 213,965)
Eastern Europe	172.55	403,942 (171,590 - 632,148)	85,064 (18,696 - 259,273)
Australasia	46.59	51,717 (22,024 - 83,796)	9,494 (1,206 - 32,912)
High-Income Asia Pacific	116.88	228,059 (76,233 - 393,707)	72,065 (25,605 - 160,438)
High-Income North America	501.04	622,388 (192,839 - 1,049,290)	74,391 (23,827 - 231,543)
Southern Latin America	67.12	95,356 (40,681 - 157,308)	59,665 (27,749 - 111,533)
Western Europe	462.8	754,970 (340,157 - 1,208,387)	174,652 (51,537 - 445,743)
Andean Latin America	57.79	44,847 (20,063 - 81,330)	69,638 (35,842 - 121,049)
Caribbean	32.95	12,348 (151 - 35,246)	34,570 (14,469 - 71,080)
Central Latin America	309.35	222,885 (70,525 - 407,729)	411,515 (215,732 - 682,765)
Tropical Latin America	202.77	111,559 (13,818 - 240,367)	195,964 (86,378 - 371,937)
North Africa and Middle East	893.58	1,395,481 (157,981 - 3,081,677)	2,573,685 (1,449,226 - 4,223,324)
Central Sub-Saharan Africa	263.34	32,073 (2,279 - 124,430)	310,028 (152,877 - 576,348)
Eastern Sub-Saharan Africa	876.2	202,329 (38,990 - 514,751)	722,054 (346,997 - 1,344,795)
Southern Sub-Saharan Africa	79.73	37,054 (9,274 - 74,878)	59,537 (28,428 - 111,484)
Western Sub-Saharan Africa	1,008.46	357,979 (44 - 954,814)	1,164,542 (647,697 - 1,948,436)
East Asia	884.05	2,969,276 (846,593 - 5,248,323)	4,164,303 (2,411,796 - 6,404,143)
Southeast Asia	625.36	417,039 (22,313 - 1,041,394)	943,949 (462,092 - 1,724,837)
Oceania	12.23	2,836 (134 - 9,747)	13,644 (4,901 - 31,800)
WORLD	8,995.74	10,777,327 (2,223,402 - 21,845,188)	19,539,434 (11,372,696 - 31,279,730)

Supplementary Table 4: Year 2090 mortality by Global Burden of Disease country and region for the Shared Socio-economic Pathways SSP5-8.5 scenario. For each country and region, the population is shown in millions, along with the attributable mortality to non-optimal temperature and to air pollution, with their respective 95% confidence intervals shown in parenthesis.

**Country or Region**	**Pop.**	**Non-optimal temperature**	**air pollution**
Armenia	1.27	7,325 (2,057 - 12,790)	8,517 (4,878 - 13,289)
Azerbaijan	6.69	33,227 (5,819 - 62,670)	31,996 (17,489 - 53,196)
Georgia	1.4	7,412 (2,449 - 12,905)	5,721 (2,839 - 10,312)
Kazakhstan	15.84	71,545 (21,623 - 123,377)	56,164 (30,822 - 95,645)
Kyrgyzstan	3.72	14,263 (5,369 - 23,593)	20,066 (11,423 - 31,197)
Mongolia	2.73	8,879 (2,040 - 17,820)	13,273 (7,183 - 23,312)
Tajikistan	2.95	11,161 (1,670 - 22,650)	16,080 (9,405 - 25,620)
Turkmenistan	4.22	18,034 (712 - 34,775)	27,490 (17,614 - 39,634)
Uzbekistan	19.28	130,761 (16,552 - 239,508)	197,590 (128,723 - 283,325)
Albania	1.98	8,745 (1,686 - 16,347)	5,525 (2,541 - 10,449)
Bosnia and Herzegovina	2.15	10,013 (3,421 - 18,674)	8,702 (4,221 - 16,076)
Bulgaria	4.83	34,293 (11,927 - 58,290)	22,229 (11,028 - 39,975)
Croatia	2.94	13,919 (4,857 - 23,957)	7,189 (3,229 - 14,073)
Czech Republic	16.34	37,491 (14,910 - 62,134)	20,705 (8,788 - 41,686)
Hungary	10.63	28,582 (10,821 - 47,696)	14,322 (6,062 - 28,842)
Rep. of Macedonia	2.0	15,413 (5,620 - 26,646)	14,447 (8,221 - 23,365)
Montenegro	0.42	1,915 (597 - 3,533)	1,271 (592 - 2,417)
Poland	38.06	91,102 (35,995 - 147,677)	48,514 (20,872 - 94,988)
Romania	10.19	62,716 (21,736 - 105,446)	23,866 (9,579 - 49,870)
Serbia	7.6	51,126 (17,670 - 89,497)	31,087 (14,841 - 58,763)
Slovakia	6.6	17,119 (6,624 - 28,601)	8,863 (3,640 - 18,074)
Slovenia	3.25	6,168 (2,085 - 11,454)	2,835 (1,184 - 6,100)
Belarus	5.61	22,581 (8,342 - 37,115)	5,763 (1,084 - 15,917)
Estonia	1.58	3,266 (1,460 - 5,228)	73 (0 - 973)
Latvia	1.28	4,944 (2,172 - 7,859)	1,309 (356 - 3,319)
Lithuania	1.57	5,788 (2,330 - 9,362)	1,303 (223 - 3,713)
Rep. of Moldova	0.68	4,868 (1,611 - 8,112)	1,340 (382 - 3,243)
Russian Federation	111.37	483,212 (182,230 - 768,814)	115,821 (31,603 - 305,593)
Ukraine	30.57	159,253 (53,094 - 265,207)	51,595 (16,124 - 121,102)
Australia	60.55	50,112 (13,808 - 89,735)	15,130 (3,055 - 44,098)
New Zealand	9.06	10,861 (5,742 - 16,486)	2,660 (567 - 7,224)

Brunei Darussalam	0.42	1,602 (189 - 3,453)	593 (163 - 1,497)
Japan	104.58	150,447 (23,105 - 275,222)	47,899 (18,297 - 99,962)
Singapore	5.66	13,557 (136 - 28,284)	5,495 (2,486 - 10,370)
Republic of Korea	44.26	113,398 (36,798 - 204,271)	61,922 (26,125 - 123,771)
Canada	83.14	112,261 (41,455 - 186,458)	6,294 (463 - 32,322)
United States of America	664.46	804,745 (184,818 - 1,414,666)	229,884 (77,481 - 545,320)
Argentina	35.66	65,383 (8,119 - 131,546)	48,700 (22,819 - 90,492)
Chile	14.03	39,008 (20,727 - 58,550)	23,505 (11,762 - 41,133)
Uruguay	1.81	3,325 (472 - 6,584)	2,821 (1,257 - 5,460)
Andorra	0.0	0 (0 - 0)	0 (0 - 0)
Austria	12.52	25,195 (9,281 - 43,232)	10,435 (4,130 - 22,025)
Belgium	19.55	30,602 (14,831 - 47,962)	12,593 (5,032 - 26,030)
Cyprus	1.19	5,672 (0 - 13,204)	3,963 (1,864 - 7,365)
Denmark	8.9	11,833 (5,981 - 18,308)	2,782 (579 - 7,532)
Finland	9.72	13,902 (6,576 - 22,083)	243 (0 - 3,148)
France	114.3	122,050 (54,517 - 196,444)	50,106 (20,096 - 103,708)
Germany	100.73	189,958 (77,990 - 316,903)	71,547 (25,148 - 159,432)
Greece	12.75	26,786 (2,027 - 51,655)	12,592 (5,794 - 23,828)
Greenland	0.03	70 (35 - 112)	0 (0 - 0)
Iceland	0.72	879 (407 - 1,414)	82 (0 - 348)
Ireland	10.23	19,413 (11,194 - 28,057)	3,742 (786 - 9,958)
Israel	29.18	17,747 (0 - 39,398)	17,937 (9,228 - 30,344)
Italy	70.22	121,831 (26,624 - 221,349)	76,210 (37,331 - 135,285)
San Marino	0.0	0 (0 - 0)	0 (0 - 0)
Luxembourg	1.24	2,073 (868 - 3,439)	835 (310 - 1,823)
Malta	0.0	0 (0 - 0)	0 (0 - 0)
Netherlands	24.77	37,319 (19,037 - 57,133)	13,577 (4,978 - 29,242)
Norway	12.33	16,090 (8,503 - 23,915)	1,512 (108 - 6,015)
Portugal	14.18	23,480 (5,862 - 42,622)	15,676 (6,902 - 30,587)
Spain	64.55	103,588 (22,065 - 190,302)	51,500 (21,890 - 100,382)
Sweden	21.79	25,613 (12,139 - 40,821)	1,645 (42 - 9,214)
Switzerland	13.36	19,997 (7,996 - 33,157)	8,394 (3,214 - 17,694)
United Kingdom	119.86	187,227 (107,365 - 269,706)	56,445 (19,977 - 123,112)
Bolivia	8.85	15,688 (4,561 - 33,895)	29,195 (15,319 - 50,686)
Ecuador	14.13	25,127 (8,379 - 46,718)	34,192 (18,552 - 55,252)
Peru	14.65	17,296 (5,939 - 32,893)	28,013 (15,460 - 46,572)
Antigua and Barbuda	0.0	0 (0 - 0)	0 (0 - 0)
Bahamas	0.01	10 (0 - 26)	5 (2 - 10)

Barbados	0.0	0 (0 - 0)	0 (0 - 0)
Belize	0.25	450 (0 - 1,022)	534 (274 - 910)
Bermuda	0.0	0 (0 - 0)	0 (0 - 0)
Cuba	3.9	9,503 (62 - 19,706)	6,182 (2,650 - 12,132)
Dominica	0.0	0 (0 - 0)	0 (0 - 0)
Dominican Republic	7.46	6,095 (252 - 14,344)	11,001 (4,967 - 21,334)
Grenada	0.0	0 (0 - 0)	0 (0 - 0)
Guyana	0.23	915 (82 - 1,883)	1,226 (692 - 1,971)
Haiti	7.37	13,040 (15 - 37,578)	20,271 (7,862 - 44,412)
Jamaica	0.83	639 (16 - 1,670)	2,161 (1,124 - 3,591)
Puerto Rico	1.01	858 (22 - 2,201)	1,025 (320 - 2,351)
United States Virgin Islands	0.0	0 (0 - 0)	0 (0 - 0)
Saint Kitts and Nevis	0.0	0 (0 - 0)	0 (0 - 0)
Saint Lucia	0.0	0 (0 - 0)	0 (0 - 0)
Saint Vincent and Grenadines	0.0	0 (0 - 0)	0 (0 - 0)
Suriname	0.46	897 (108 - 1,856)	1,232 (668 - 2,036)
Trinidad and Tobago	0.56	1,140 (248 - 2,201)	2,299 (1,259 - 3,690)
Colombia	47.56	58,318 (11,083 - 115,612)	120,032 (65,520 - 189,032)
Costa Rica	5.6	3,817 (232 - 9,949)	14,265 (7,722 - 22,742)
El Salvador	2.04	2,032 (0 - 4,822)	5,764 (3,254 - 8,957)
Guatemala	16.51	19,534 (1,296 - 44,602)	55,181 (33,035 - 85,052)
Honduras	7.7	7,392 (6 - 21,672)	29,606 (14,776 - 52,712)
Mexico	97.33	148,602 (33,087 - 276,835)	195,233 (100,043 - 322,048)
Nicaragua	3.23	7,048 (234 - 15,156)	9,235 (5,006 - 15,123)
Panama	3.99	4,215 (342 - 8,951)	7,758 (3,968 - 12,974)
Venezuela	30.73	42,497 (702 - 96,451)	86,364 (49,195 - 133,613)
Brazil	153.53	194,131 (2,543 - 428,581)	260,202 (120,494 - 472,533)
Paraguay	6.99	7,663 (2 - 18,436)	11,189 (5,285 - 20,675)
Afghanistan	59.43	114,683 (17,225 - 264,222)	234,989 (119,366 - 405,417)
Algeria	35.87	168,406 (6,955 - 376,120)	134,408 (62,436 - 261,301)
Bahrain	0.67	3,230 (0 - 7,446)	0 (0 - 0)
Egypt	96.91	277,033 (12,120 - 653,816)	513,418 (298,121 - 824,716)
Iran	65.41	284,202 (40,498 - 543,250)	366,499 (223,216 - 541,734)
Iraq	59.55	171,381 (1,601 - 412,507)	344,067 (203,547 - 538,972)
Jordan	13.84	37,953 (0 - 90,990)	57,635 (30,868 - 96,231)
Kuwait	3.94	9,210 (0 - 21,272)	15,277 (9,610 - 21,822)
Lebanon	3.25	9,424 (541 - 21,334)	8,527 (4,023 - 15,647)
Libya	7.51	12,824 (18 - 32,755)	17,016 (7,890 - 32,426)

Morocco	20.08	50,019 (4,226 - 119,944)	82,189 (40,253 - 149,106)
State of Palestine	2.92	7,918 (0 - 18,799)	9,439 (4,606 - 17,211)
Oman	3.62	14,606 (67 - 33,406)	25,441 (15,606 - 38,681)
Qatar	2.24	13,782 (0 - 32,816)	28,585 (17,531 - 43,769)
Saudi Arabia	55.45	170,419 (119 - 405,048)	374,233 (233,456 - 552,118)
Sudan	54.81	117,809 (59 - 307,518)	221,414 (115,480 - 379,574)
Syrian Arab Republic	26.27	132,682 (442 - 285,842)	158,236 (89,127 - 259,222)
Tunisia	9.02	28,707 (280 - 66,255)	29,354 (13,873 - 55,246)
United Arab Emirates	16.86	113,487 (0 - 304,785)	198,184 (118,224 - 318,510)
Turkey	70.39	190,217 (40,566 - 397,533)	171,818 (79,990 - 322,469)
Yemen	47.07	52,933 (4,970 - 131,501)	175,931 (94,732 - 298,332)
Bangladesh	127.97	382,057 (0 - 1,005,887)	1,013,460 (597,779 - 1,689,260)
Bhutan	1.06	1,922 (560 - 4,227)	4,052 (1,960 - 7,485)
India	1,254.02	3,046,399 (12,905 - 7,387,363)	7,548,674 (4,874,679 - 10,938,261)
Nepal	38.07	70,906 (4,823 - 195,024)	236,270 (126,630 - 384,796)
Pakistan	219.74	450,534 (3,309 - 1,195,348)	1,245,752 (757,196 - 1,935,592)
South Asia	1,640.85	3,951,819 (21,598 - 9,787,849)	10,048,209 (6,358,244 - 14,955,394)
Angola	43.49	20,043 (6 - 63,543)	83,375 (39,292 - 158,679)
Central African Republic	5.92	6,505 (0 - 20,403)	20,893 (9,917 - 39,416)
Congo	7.86	8,966 (69 - 25,522)	29,387 (14,859 - 52,748)
Dem. Rep. of the Congo	146.44	92,618 (1,026 - 273,122)	373,080 (192,350 - 673,112)
Equatorial Guinea	1.15	574 (0 - 1,935)	3,015 (1,446 - 5,674)
Gabon	1.44	2,394 (2 - 6,409)	4,824 (2,317 - 8,839)
Burundi	18.17	4,594 (83 - 18,751)	64,705 (34,683 - 110,445)
Comoros	0.0	0 (0 - 0)	0 (0 - 0)
Djibouti	0.92	1,884 (0 - 5,207)	2,042 (1,042 - 3,611)
Eritrea	10.98	14,265 (0 - 47,198)	30,839 (14,536 - 57,480)
Ethiopia	139.67	101,648 (15,327 - 252,752)	292,087 (147,897 - 516,541)
Kenya	67.87	29,498 (4,454 - 75,451)	119,015 (65,197 - 197,790)
Madagascar	37.8	31,763 (3,081 - 81,109)	80,409 (37,328 - 152,692)
Malawi	40.19	21,054 (2 - 62,704)	59,005 (29,180 - 107,057)
Mozambique	37.65	34,086 (0 - 96,904)	56,363 (26,013 - 108,108)
Rwanda	21.34	7,425 (802 - 22,548)	66,777 (35,006 - 114,982)

Somalia	7.95	9,536 (0 - 26,882)	9,076 (3,704 - 19,719)
South Sudan	12.76	19,886 (0 - 54,511)	22,650 (10,576 - 42,482)
United Republic of Tanzania	85.05	34,128 (143 - 110,906)	159,637 (81,596 - 280,492)
Uganda	93.44	16,302 (228 - 68,280)	189,934 (99,171 - 327,179)
Zambia	28.4	14,178 (0 - 42,980)	46,079 (22,886 - 83,561)
Botswana	2.49	4,121 (0 - 12,232)	5,683 (2,299 - 12,200)
Lesotho	2.04	3,040 (1,039 - 6,186)	4,359 (1,941 - 8,640)
Namibia	2.91	5,011 (34 - 14,364)	8,390 (3,911 - 16,035)
South Africa	53.78	53,331 (5,863 - 111,747)	104,267 (56,554 - 172,358)
Swaziland	1.36	964 (17 - 2,797)	2,484 (1,004 - 5,298)
Zimbabwe	6.02	5,478 (1 - 15,735)	9,875 (4,357 - 19,942)
Benin	16.19	35,453 (0 - 89,352)	52,045 (29,511 - 84,490)
Burkina Faso	34.68	67,188 (0 - 169,537)	79,584 (44,427 - 130,450)
Cameroon	29.66	29,346 (1 - 81,811)	105,354 (59,402 - 171,582)
Cape Verde	0.0	0 (0 - 0)	0 (0 - 0)
Chad	23.42	34,224 (0 - 90,540)	50,699 (27,016 - 87,621)
Cote d'Ivoire	21.32	46,861 (0 - 118,301)	58,209 (29,285 - 104,066)
Gambia	2.54	5,003 (35 - 12,532)	8,089 (4,478 - 13,365)
Ghana	38.75	101,734 (0 - 247,504)	130,149 (72,607 - 211,724)
Guinea	8.98	13,101 (0 - 34,597)	23,024 (12,073 - 39,677)
Guinea-Bissau	1.93	4,205 (11 - 10,664)	5,773 (3,115 - 9,825)
Liberia	12.26	17,764 (22 - 44,376)	26,286 (12,991 - 47,638)
Mali	31.95	50,897 (0 - 132,627)	81,781 (44,754 - 134,983)
Mauritania	5.1	9,297 (0 - 24,090)	15,693 (9,033 - 25,155)
Niger	49.75	52,005 (0 - 139,427)	98,067 (54,735 - 162,154)
Nigeria	411.99	427,849 (0 - 1,037,662)	953,349 (564,561 - 1,520,093)
Sao Tome and Principe	0.0	0 (0 - 0)	0 (0 - 0)
Senegal	10.89	18,502 (10 - 46,726)	31,454 (17,898 - 50,814)
Sierra Leone	10.7	14,519 (0 - 36,673)	23,399 (11,663 - 42,031)
Togo	9.25	23,506 (0 - 59,264)	31,963 (17,687 - 52,970)
China	763.59	4,430,087 (589,174 - 8,487,145)	6,221,789 (3,618,825 - 9,424,890)
Dem. People's Rep. of Korea	15.84	86,251 (27,596 - 173,845)	52,425 (21,257 - 114,195)
Taiwan	19.16	31,098 (1,455 - 70,160)	32,388 (15,569 - 58,040)
Cambodia	10.0	29,493 (0 - 71,889)	16,594 (6,802 - 35,708)
Indonesia	180.01	371,406 (43,983 - 766,969)	479,754 (250,463 - 838,648)
Lao People's Dem. Rep.	5.48	9,892 (0 - 27,149)	15,319 (6,915 - 30,656)
Malaysia	35.48	65,924 (5,621 - 142,357)	65,607 (29,834 - 127,282)
Maldives	0.0	0 (0 - 0)	0 (0 - 0)

Mauritius	0.68	686 (0 - 1,661)	1,689 (853 - 2,927)
Myanmar	27.24	72,677 (73 - 183,041)	131,030 (67,498 - 230,621)
Philippines	105.7	172,551 (2,731 - 373,443)	214,874 (118,803 - 349,039)
Seychelles	0.0	0 (0 - 0)	0 (0 - 0)
Sri Lanka	11.45	26,535 (1,634 - 59,203)	30,085 (14,607 - 54,582)
Thailand	50.86	105,911 (92 - 258,594)	87,471 (39,279 - 170,730)
Timor-Leste	0.59	164 (0 - 549)	441 (165 - 1,011)
Viet Nam	67.55	212,237 (353 - 553,135)	207,532 (87,705 - 409,206)
American Samoa	0.0	0 (0 - 0)	0 (0 - 0)
Micronesia	0.0	0 (0 - 0)	0 (0 - 0)
Fiji	0.27	250 (0 - 742)	400 (93 - 1,101)
Guam	0.0	0 (0 - 0)	0 (0 - 0)
Kiribati	0.0	0 (0 - 0)	0 (0 - 0)
Nauru	0.0	0 (0 - 0)	0 (0 - 0)
Niue	0.0	0 (0 - 0)	0 (0 - 0)
Northern Mariana Islands	0.0	0 (0 - 0)	0 (0 - 0)
Palau	0.0	0 (0 - 0)	0 (0 - 0)
Marshall Islands	0.0	0 (0 - 0)	0 (0 - 0)
Papua New Guinea	8.49	6,513 (548 - 18,521)	17,047 (6,387 - 38,460)
Western Samoa	0.0	0 (0 - 0)	0 (0 - 0)
Solomon Islands	0.17	174 (3 - 506)	331 (117 - 796)
Tonga	0.0	0 (0 - 0)	0 (0 - 0)
Tokelau	0.0	0 (0 - 0)	0 (0 - 0)
Vanuatu	0.05	26 (0 - 109)	78 (25 - 202)
Central-Eastern Europe and Central Asia	317.75	1,365,122 (447,480 - 2,291,734)	763,655 (374,945 - 1,434,066)
High-Income	1,685.75	2,366,024 (728,668 - 4,076,475)	856,720 (331,883 - 1,848,722)
Latin America and Caribbean	434.92	586,904 (69,211 - 1,237,060)	932,165 (477,445 - 1,580,408)
North Africa and Middle East	655.12	1,980,923 (129,688 - 4,527,162)	3,166,661 (1,781,954 - 5,172,504)
South Asia	1,640.85	3,951,819 (21,598 - 9,787,849)	10,048,209 (6,358,244 - 14,955,394)
Sub-Saharan Africa	1,596.46	1,494,744 (32,258 - 3,895,858)	3,623,167 (1,954,296 - 6,183,717)
Southeast-East Asia and Oceania	1,302.61	5,621,877 (673,264 - 11,189,017)	7,574,854 (4,285,196 - 11,888,093)

Central Asia	58.1	302,608 (58,290 - 550,086)	376,897 (230,376 - 575,530)
Central Europe	106.99	378,601 (137,950 - 639,950)	209,554 (94,797 - 404,676)
Eastern Europe	152.66	683,913 (251,240 - 1,101,699)	177,204 (49,772 - 453,860)
Australasia	69.61	60,973 (19,550 - 106,222)	17,790 (3,622 - 51,322)
High-Income Asia Pacific	154.92	279,003 (60,228 - 511,230)	115,910 (47,070 - 235,600)
High-Income North America	747.61	917,006 (226,273 - 1,601,125)	236,178 (77,943 - 577,643)
Southern Latin America	51.5	107,716 (29,317 - 196,680)	75,027 (35,838 - 137,084)
Western Europe	662.11	1,001,325 (393,300 - 1,661,218)	411,816 (167,410 - 847,073)
Andean Latin America	37.63	58,111 (18,879 - 113,506)	91,401 (49,332 - 152,510)
Caribbean	22.06	33,545 (804 - 82,486)	45,936 (19,816 - 92,437)
Central Latin America	214.7	293,454 (46,983 - 594,050)	523,438 (282,519 - 842,252)
Tropical Latin America	160.53	201,794 (2,545 - 447,017)	271,390 (125,778 - 493,209)
North Africa and Middle East	655.12	1,980,923 (129,688 - 4,527,162)	3,166,661 (1,781,954 - 5,172,504)
Central Sub-Saharan Africa	206.3	131,100 (1,103 - 390,934)	514,574 (260,181 - 938,468)
Eastern Sub-Saharan Africa	602.2	340,247 (24,121 - 966,181)	1,198,616 (608,815 - 2,122,139)
Southern Sub-Saharan Africa	68.6	71,945 (6,954 - 163,061)	135,059 (70,066 - 234,472)
Western Sub-Saharan Africa	719.37	951,453 (80 - 2,375,682)	1,774,917 (1,015,234 - 2,888,639)
East Asia	798.58	4,547,436 (618,225 - 8,731,150)	6,306,602 (3,655,650 - 9,597,124)
Southeast Asia	495.06	1,067,477 (54,488 - 2,437,990)	1,250,395 (622,925 - 2,250,410)
Oceania	8.98	6,964 (551 - 19,878)	17,856 (6,621 - 40,559)
WORLD	7,614.3	17,336,315 (2,100,711 - 36,934,995)	26,933,042 (15,548,396 - 43,004,864)
